# Polymers for Detecting or Protecting Against Chemical Warfare Agents: Recent Advances and Future Perspectives

**DOI:** 10.3390/polym18192362

**Published:** 2026-09-28

**Authors:** Xiaotong Yue, Tingting Wang, Aishuo Yu, Xiaopeng Li, Min Zhang, Xiaohui Zheng, Xinglan Wang, Yingru Li, Xiaoshan Yan, Li Li, Wei He

**Affiliations:** 1 State Key Laboratory of Chemistry for NBC Hazards Protection, Beijing 102205, China; 2 School of Materials Science and Engineering, University of Science and Technology Beijing, Beijing 100083, China; 3 Key Laboratory of Green Manufacturing of Super-Light Elastomer Materials of State Ethnic Affairs Commission, Hubei Minzu University, Enshi 445000, China; 2020030@hbmzu.edu.cn; 4 College of Intelligent Systems Science and Engineering, Hubei Minzu University, Enshi 445000, China

**Keywords:** CWAs, polymers, detection, protection, degradation

## Abstract

The persistent threat of chemical warfare agents (CWAs) drives the need for advanced detection and protective technologies. Polymers, with their tunable structures, ease of functionalization, and lightweight nature, have emerged as a versatile platform in this field. This review systematically summarizes recent progress in polymer-based materials for CWA sensing and protection, with emphasis on interaction mechanisms and structure–property relationships. In the realm of sensing, the working principles of polymer-based systems are rooted in electron transfer, hydrogen bonding, fluorescence quenching, and colorimetric response. Conductive polymers enable chemiresistive detection through charge transfer; hydrogen-bond acidic polymers provide selective recognition of organophosphorus agents; conjugated polymers exploit fluorescence quenching via the “molecular wire” effect; and polydiacetylenes offer visible color changes for naked-eye detection. Representative materials and their performance metrics are critically compared. For protection and decontamination, current polymer systems are designed around four synergistic mechanisms: barrier action, physical adsorption, filtration, and catalytic degradation. Barrier layers based on crosslinked networks or graphene/MOF composites suppress agent permeation while maintaining breathability. Porous polymers such as polymers of intrinsic microporosity (PIMs) and coordination polymers provide high-capacity adsorption through tailored surface functionality. Electrospun nanofiber membranes effectively filter aerosolized agents with low air resistance. Catalytic composites incorporating Zr-MOFs or single-atom catalysts enable hydrolysis of nerve agents and oxidation of blister agents under ambient conditions, with recent advances achieving self-buffering and solid-state operation. Despite significant advances, challenges remain in selectivity, environmental stability, and balancing protection with wearer comfort. Future directions point toward multifunctional systems that integrate detection, protection, and self-detoxification within wearable polymer platforms for next-generation chemical defense.

## 1. Introduction

Chemical warfare agents (CWAs) are highly toxic chemical compounds designed for use in military operations and terrorist activities. Since their large-scale deployment during World War I, CWAs have posed persistent threats to human life, social stability, and world peace [1]. Based on their physiological effects, CWAs are generally categorized into four main classes: nerve agents (e.g., sarin, soman, tabun, and VX), blister agents (e.g., sulfur mustard and lewisite), blood agents (e.g., hydrogen cyanide), and choking agents (e.g., phosgene and chlorine) [2,3]. Exposure to these agents can result in severe health consequences, including respiratory spasms, edema, increased secretions, breathing difficulties, and in many cases, death.

The threat of CWAs is not merely historical but remains a pressing contemporary concern. Beyond state-level military arsenals, non-state actors have demonstrated both the intent and capability to deploy chemical weapons. The Islamic State of Iraq and the Levant (ISIL) has been identified by the Organisation for the Prohibition of Chemical Weapons (OPCW) as the perpetrator of multiple chemical attacks in Syria and Iraq. Notably, in September 2015 in Marea, Syria, ISIL units deployed sulfur mustard, and over twenty chemical attacks by the group have been reported in the region [4]. These incidents underscore the urgent necessity of developing reliable technologies for both the detection of and protection against CWAs [5]. Recognizing this imperative, major nations including the United States, Russia, the European Union member states, and China have invested substantial efforts in advancing research and development in chemical defense technologies.

Detection technologies for CWAs can be broadly divided into two categories according to their application scenarios. The first category comprises precise, laboratory-based analytical instrumental methods, such as gas chromatography–mass spectrometry (GC–MS) and other high-resolution benchtop instruments, which offer excellent sensitivity and specificity but require well-equipped facilities and trained personnel [6,7]. The second category encompasses rapid, on-site detection techniques suitable for field deployment and real-time qualitative or semi-quantitative analysis. These include ion mobility spectrometry (IMS), electrochemical sensors, fluorescence-based sensors, colorimetric methods, chemiresistive sensors, and emerging technologies such as quartz-enhanced photoacoustic spectroscopy (QEPAS) [8]. Portable devices based on these principles enable first responders and military personnel to obtain timely threat assessments in contaminated environments.

Protection against CWAs has historically relied on barrier and filtration materials. Early protective gear, such as natural rubber garments and activated carbon-impregnated masks, provided physical isolation from chemical agents [9]. Over time, the development of advanced filtration media—including those used in respirators and collective protection systems—has significantly enhanced the efficacy of personal protective equipment [10]. Polymeric materials have played a central role in this evolution, owing to their unique combination of lightweight flexibility, physiological comfort, and adaptable barrier properties.

Polymers represent both a traditional and a burgeoning class of materials for CWA detection and protection [11]. In sensing applications, polymers can serve as selective recognition layers—for example, hydrogen-bond acidic polymers exhibit high affinity for organophosphorus nerve agents, while molecularly imprinted polymers (MIPs) offer biomimetic recognition capabilities for target CWA molecules [12]. In protective applications, polymers function as barrier coatings, adsorbents, and catalytic degradation media [13]. Beyond their intrinsic chemical functionality, polymers can also serve as versatile matrices for integrating inorganic, carbon-based, and nanostructured components, enabling complementary functions through polymer–component interactions and interfacial engineering [14]. Recent advances have been driven by multilevel structural engineering, encompassing molecular-level design (e.g., functional group tuning, crosslinking density control, and chain architecture manipulation) and composite-level design (e.g., polymer–metal–organic framework composites, polymer–carbon composites, and fabric-based functionalized composites) [15,16,17]. These strategies have yielded materials with enhanced sensitivity, selectivity, barrier performance, and even self-detoxifying capabilities.

Compared with previous reviews, the present work is distinguished by its explicitly polymer-centered and structure–property–performance-oriented perspective. Recent reviews have addressed important aspects of CWA-related technologies, including fluorescent probes and fluorescent/colorimetric chemosensors for CWA detection [18], functionalized reactive polymers for CWA removal and detoxification [19], and broader CWA decontamination strategies encompassing multiple classes of materials [2]. In contrast, this review integrates both CWA sensing and protection/decontamination within a unified polymer-materials framework, while systematically connecting molecular interactions and polymer structure with functional performance. Particular emphasis is placed on how polymer chain architecture, crosslinking, free volume, pore structure, surface functionality, and polymer–filler interfaces govern sensing, barrier, adsorption, filtration, and catalytic performance. The review also explicitly distinguishes the functional role of polymers in polymer-containing composites, particularly when inorganic or carbon-based components provide the dominant active function. In addition, practical considerations—including scalability, cost, durability, environmental stability, sorption-induced swelling and plasticization, and the distinction between actual CWAs and CWA simulants—are critically discussed. This integrated framework is intended to complement existing reviews by providing a polymer-focused materials-design perspective that links molecular interactions, structural characteristics, material properties, functional performance, and practical applicability.

## 2. Mechanisms of the Interactions Between CWAs and Polymeric Materials

The interactions between polymeric materials and CWAs constitute the theoretical foundation for the detection, protection, and decontamination of CWAs. Representative CWAs and their commonly used simulants, together with their corresponding chemical structures, are illustrated in Figure 1. Depending on the intended application, these interactions can be broadly classified into two categories: those employed for CWAs detection and those utilized for CWAs protection. Although both are based on the interfacial interactions between polymeric materials and CWAs molecules, they differ fundamentally in their objectives and mechanisms of action. For sensing materials, the primary objective is to exploit electron transfer, hydrogen bonding, charge redistribution, or intermolecular energy transfer between specific functional groups or conjugated structures within the polymer and CWAs molecules, thereby inducing measurable electrical, optical, or colorimetric signal changes for the highly sensitive recognition of target analytes [19]. Fundamentally, this process can be regarded as a “molecular recognition–signal transduction” mechanism. In contrast, protective materials are designed to reduce the risk of CWAs exposure to the human body. Their protective mechanisms mainly involve suppressing the diffusion of CWAs through polymer networks, physically adsorbing CWAs molecules using porous materials, intercepting aerosol particles with filtration media, and catalytically degrading or transforming CWAs through active components. Essentially, these processes can be summarized as a “barrier–removal–degradation” mechanism.

### 2.1. Mechanisms of Polymer-Based Detection of CWAs

Polymer-based sensing materials primarily rely on the molecular recognition interactions between polymers and CWAs molecules, converting molecular-scale interactions into detectable electrical or optical signals. Since nerve agents (e.g., sarin (GB), soman (GD), and VX) and their simulants, such as dimethyl methylphosphonate (DMMP), generally possess highly polar phosphoryl groups, while blister agents (e.g., sulfur mustard (HD) and 2-chloroethyl ethyl sulfide (CEES)) contain sulfur atoms and haloalkyl moieties, they are capable of undergoing various noncovalent interactions with hydroxyl, amino, carboxyl, and amide groups, as well as π-conjugated systems within polymeric materials, thereby generating detectable response signals. At present, the detection of CWAs using polymer-based sensing materials is mainly based on four mechanisms: electron transfer, hydrogen bonding, fluorescence quenching, and colorimetric response (Figure 2).

(1) Electron transfer

Electron transfer is the most prevalent signal transduction mechanism in conductive polymer- and semiconducting polymer-based sensors. Fundamentally, when a polymer comes into contact with a target molecule, differences in their Fermi energy levels or highest occupied molecular orbital (HOMO) and lowest unoccupied molecular orbital (LUMO) drive electron redistribution toward a lower-energy state. This process alters the concentration and mobility of charge carriers within the material, ultimately leading to changes in resistance, current, or electrical conductivity [20]. In π-conjugated conductive polymers, such as polyaniline (PANI), polypyrrole (PPy), polythiophene (PTh), and poly(3,4-ethylenedioxythiophene) (PEDOT), the polymer backbones consist of continuous π-electron conjugated systems, in which electrons exhibit a high degree of delocalization [21]. Upon adsorption of target molecules with strong electron-donating or electron-accepting characteristics, intermolecular charge transfer occurs, thereby modulating the doping level and charge carrier concentration of the polymer. For example, electron-accepting molecules can extract electrons from the polymer, resulting in an increased hole concentration and a corresponding decrease in the resistance of p-type conductive polymers. In contrast, electron-donating molecules inject electrons into the polymer, neutralizing a portion of the holes, reducing the charge carrier concentration, and consequently increasing the electrical resistance [22]. In the case of the nerve agent simulant DMMP, its highly polar phosphoryl group can induce electron redistribution upon interaction with the surface of conductive polymers or polymer-based composites, thereby modifying the interfacial energy band structure and enabling the electrical detection of the target molecule.

(2) Hydrogen-bonding interactions

Hydrogen bonding is one of the most important molecular recognition mechanisms by which polymers recognize nerve agents and their simulants. Fundamentally, it involves the formation of directional noncovalent interactions between hydrogen bond donors in the polymer and hydrogen bond acceptors in the target molecules. Nerve agents and their simulants, including DMMP, GB, GD, and VX, generally contain highly polar phosphoryl groups, in which the oxygen atom possesses a high electron density and serves as an excellent hydrogen bond acceptor. When polymers contain functional groups such as hydroxyl (–OH), amino (–NH_2_), amide (–CONH–), urea (–NHCONH–), or thiourea moieties [23], stable hydrogen-bonded complexes can be formed with the P=O group, enabling the highly selective recognition of target molecules. The formation of hydrogen bonds induces local electron density redistribution within the polymer, leading to changes in molecular conformation, dipole moment, and energy level alignment, which subsequently trigger electron transfer, variations in electrical conductivity, fluorescence quenching, or changes in the absorption spectrum [24]. Therefore, in many polymer-based sensing systems, hydrogen bonding is not the ultimate source of the sensing signal but rather serves as a prerequisite for achieving highly selective molecular recognition.

(3) Fluorescence quenching

Fluorescence quenching fundamentally occurs when the radiative decay of excited-state electrons is suppressed following the recognition of target molecules by the polymer, resulting in a reduction in fluorescence intensity, a shortened fluorescence lifetime, or a shift in the emission wavelength [25]. Depending on the quenching mechanism, fluorescence quenching can be broadly classified into two categories: dynamic quenching and static quenching. Dynamic quenching arises from collisional interactions between excited-state fluorophores and quencher molecules, leading to the dissipation of excitation energy through non-radiative pathways. In contrast, static quenching results from the formation of stable ground-state complexes, thereby reducing the number of fluorescent molecules available for excitation. In polymer-based sensing systems for CWAs, fluorescence responses are generally governed by mechanisms such as photoinduced electron transfer (PET), intramolecular charge transfer (ICT), or fluorescence resonance energy transfer (FRET) [26,27,28]. Upon binding of the target molecule to the polymer, the energy level alignment between electron donors and acceptors is altered, resulting in changes to the electron transfer pathway and rapid deactivation of the excited state, thereby inducing fluorescence quenching. For conjugated polymers, the presence of a continuous π-electron delocalized backbone enables the rapid migration of excitation energy along the polymer chain [29]. Consequently, the binding of a single target molecule can influence exciton migration throughout the entire polymer chain, giving rise to a pronounced “molecular wire” effect that amplifies the sensing signal and affords detection sensitivities substantially higher than those of conventional small-molecule fluorescent probes.

(4) Colorimetric response

Colorimetric sensing is a detection mechanism that enables the visual identification of target molecules through color changes in polymeric materials induced by their interactions with analytes. Fundamentally, these color variations arise from changes in the electronic structure or aggregation state of the polymer [30], which alter its optical absorption spectrum and consequently produce observable changes in visible color. Polymer-based colorimetric sensing generally involves three principal mechanisms. First, the formation of complexes between the polymer and target molecules modifies the degree of electron delocalization, thereby changing the energy levels associated with π–π* electronic transitions [31]. Second, target molecules may induce polymer aggregation or disaggregation, leading to changes in intermolecular interactions. Third, the target molecules may directly participate in chemical reactions to generate new chromophoric species. For conductive polymers, color changes are typically accompanied by variations in the oxidation state or doping level of the polymer. For example, polyaniline exhibits distinct colors, including green, blue, and violet, under different oxidation states. When the target molecule induces electron redistribution, the absorption bands of the polymer shift, resulting in pronounced color changes.

In summary, the detection of CWAs using polymer-based sensing materials primarily relies on four mechanisms: electron transfer, hydrogen bond-mediated molecular recognition, fluorescence quenching, and colorimetric response. Among these, hydrogen bonding determines the molecular recognition capability of polymers toward CWAs, whereas electron transfer and energy transfer are responsible for converting molecular recognition events into measurable output signals. In recent years, the integration of multiple sensing mechanisms has emerged as an important strategy for enhancing the sensitivity, selectivity, and anti-interference capability of polymer-based CWAs sensors.

### 2.2. Mechanisms of Polymer-Based Protection of CWAs

The primary objective of polymer-based protective materials is to prevent the permeation of CWAs through the material and thereby reduce the risk of human exposure. Their protective mechanisms generally include diffusion barrier effects, physical adsorption, filtration and interception, and catalytic degradation. In recent years, with the rapid development of porous polymers, metal–organic frameworks (MOFs), covalent organic frameworks (COFs), hydrogen-bonded organic frameworks (HOFs), and polymer-based nanocomposites, CWAs protective materials have gradually evolved from traditional “passive barrier” systems toward “active adsorption–catalytic degradation” systems (Figure 3).

(1) Barrier

Barrier protection is the most fundamental mechanism by which polymeric materials protect against CWAs. Fundamentally, it relies on the continuous and dense polymer network to restrict the migration of CWAs molecules, thereby reducing their diffusion rate through the material. For liquid or gaseous CWAs, permeation through polymers generally follows the solution–diffusion model, in which CWAs molecules are first adsorbed onto and dissolved in the polymer surface, and subsequently diffuse through the free volume between polymer chains under the driving force of a concentration gradient before eventually being released from the opposite side of the material. Therefore, the barrier performance of polymers against CWAs is primarily determined by the packing arrangement of the polymer chains and the internal free volume of the polymer matrix [32]. Polymers with a high cross-linking density, high crystallinity, or rigid chain segments exhibit more compact chain packing, which significantly reduces the free volume and restricts chain mobility, thereby creating more tortuous diffusion pathways for CWAs molecules [11] and consequently decreasing their diffusion coefficient. In addition, polar functional groups within the polymer can form weak dipole–dipole interactions or hydrogen bonds with CWAs molecules, further retarding their migration through the polymer matrix to a certain extent. Fundamentally, the barrier mechanism represents a passive protection strategy that delays the permeation of CWAs rather than altering their chemical properties. Consequently, once the adsorption capacity of the polymer becomes saturated or the material is exposed to high concentrations of CWAs for prolonged periods, CWAs molecules may eventually permeate through the protective material.

(2) Physical Adsorption

Physical adsorption is an important mechanism by which polymer-based protective materials remove CWAs. Fundamentally, it involves the enrichment of CWAs molecules on the material surface or within its porous structure through noncovalent interactions, such as van der Waals forces, dipole–dipole interactions, π–π interactions, capillary condensation, and hydrogen bonding, without inducing any chemical reactions, thereby reducing their mobility in the surrounding environment. The adsorption performance is primarily determined by the specific surface area, pore size distribution, pore volume, and surface functional groups of the adsorbent. In general, a larger specific surface area provides more accessible adsorption sites; micropores (<2 nm) are favorable for increasing the adsorption capacity, whereas mesopores (2–50 nm) facilitate mass transport and accelerate the diffusion of CWAs molecules into the interior of the material [33,34]. Therefore, hierarchical porous structures, which simultaneously offer high adsorption capacity and efficient mass transfer, have become an important direction in the development of advanced adsorbent materials in recent years. For nerve agents and their simulant DMMP, the relatively large molecular dipole moment and highly polar phosphoryl group enable dipole–dipole interactions or hydrogen bonding with polar functional groups, such as hydroxyl, carboxyl, and amide groups, in polymeric materials, thereby enhancing the adsorption capacity. In the case of the blister agent HD and its simulant CEES, the sulfur-containing structure can be effectively enriched within porous polymers through van der Waals forces and hydrophobic interactions. However, physical adsorption is inherently a reversible process. When the ambient concentration decreases or the temperature increases, the adsorbed CWAs molecules may desorb from the material. Consequently, adsorption alone is insufficient to provide long-term and reliable protection against CWAs.

(3) Filtration

Filtration is an important mechanism by which polymeric fibrous materials remove CWA-containing aerosols and liquid droplets. Fundamentally, it relies on the three-dimensional fibrous network formed by polymer fibers to intercept airborne CWA-laden particles, thereby preventing their entry into the human respiratory system. Unlike the barrier mechanism, which depends on dense membrane layers, filtration is based on highly porous fibrous networks with large specific surface areas, where pollutant removal is achieved through the combined effects of physical sieving and interfacial interactions. As CWA-containing particles pass through the polymer fiber layer, larger particles are primarily captured by inertial impaction and direct interception, whereas smaller particles continuously deviate from the airflow streamlines due to Brownian diffusion, increasing the probability of contact with the fiber surface, where they are subsequently adsorbed [35]. In addition, electrostatic charges on the surface of polymer fibers can enhance the electrostatic attraction of charged particles or polar liquid droplets, thereby further improving the filtration efficiency. In recent years, polymer nanofibrous membranes fabricated by electrospinning have attracted considerable attention because their smaller fiber diameters, higher porosity, and larger specific surface areas significantly increase the probability of particle–fiber interactions, enabling enhanced filtration performance while maintaining a relatively low air-flow resistance.

(4) Catalytic Degradation

Catalytic degradation is an active protection strategy that relies on catalytically active sites to promote the hydrolysis, oxidation, or redox transformation of CWAs, converting them into less toxic or even nontoxic small-molecule products and thereby fundamentally eliminating their hazardous effects. Unlike barrier protection and physical adsorption, catalytic degradation not only reduces the concentration of CWAs in the environment but also prevents the secondary release of toxic agents caused by adsorbent saturation. Consequently, it has been widely recognized as a key development direction for next-generation intelligent protective materials. Since polymers themselves generally possess limited intrinsic catalytic activity, their degradation capability is typically introduced through chemical functionalization or by incorporating catalytically active components. For example, the homogeneous dispersion of metal oxides, MOFs, covalent organic frameworks (COFs), or nanozymes within polymer matrices [36] endows the resulting composites with both mechanical flexibility and catalytic activity. After the nerve agent simulant DMMP diffuses into the polymer matrix, it is first enriched and transported through the porous network to the catalytic active sites. Subsequently, Lewis acid sites activate the phosphoryl group, thereby increasing the electrophilicity of the phosphorus atom and facilitating the nucleophilic attack of water molecules or hydroxyl groups on the phosphoester bond. This process ultimately leads to P–O bond cleavage and the formation of less toxic phosphonic acid derivatives. For the blister agent HD and its simulant CEES, catalytically active components within the polymer promote thioether oxidation or haloalkyl hydrolysis, converting these compounds into less toxic sulfoxides, sulfones, or alcohols [37].

Overall, polymer-based protective materials achieve the transition from “delayed permeation” to “active detoxification” through the synergistic effects of barrier protection, physical adsorption, filtration, and catalytic degradation. Among these mechanisms, barrier protection and filtration primarily reduce the migration rate of CWAs, physical adsorption enhances the local enrichment of hazardous molecules, whereas catalytic degradation fundamentally eliminates their toxicity through chemical transformation. In the future, intelligent polymer-based protective materials that simultaneously combine superior barrier performance, rapid catalytic activity, and high air permeability are expected to become a major research direction in the field of chemical warfare agent protection.

## 3. Polymer-Based Detection Mechanisms and Representative Systems

As detailed in Section 2, the sensing performance of polymer materials toward CWAs is governed by four primary interaction mechanisms: electron transfer, hydrogen-bonding, fluorescence quenching, and colorimetric response. Rather than reiterating the mechanistic principles, this section focuses on representative material systems, their structure–property relationships, key performance metrics, and current limitations. For each category, we highlight the functional role of the polymer component—whether it serves as the active signal-transducing layer, the molecular-recognition layer, or a supporting matrix—and critically evaluate the design strategies that translate molecular-level interactions into detectable signals [38].

### 3.1. Electron Transfer

Electron transfer serves as the primary signal transduction mechanism in chemiresistive sensors based on conducting polymers (as described in Section 2.1).

In this section, we survey representative material systems where conductive polymers—either as the active sensing layer or as a conductive matrix for nanofillers—convert analyte-induced changes in charge carrier concentration into measurable electrical resistance signals. Particular attention is paid to the structural design strategies that enhance electron transfer efficiency and the resulting sensing performance.

Recent strategies to enhance electron transfer efficiency have focused on three design approaches: functional group engineering, nanofiller incorporation, and molecular-level structural optimization. In carboxyl-functionalized polypyrrole nanotubes, the conducting polymer backbone serves as the signal-transducing layer, while the surface carboxyl groups act as specific binding sites for organophosphorus molecules; this dual functionality enables efficient charge transfer upon analyte binding, achieving sub-ppb detection of nerve agent simulants [39].

For CNT/polythiophene composite systems, polythiophene functions as the chemiresistive matrix that undergoes conformational changes upon analyte intercalation, while CNTs provide a continuous conductive network that amplifies the electrical response through a synergistic combination of charge transfer, scattering-site introduction, and polymer chain rearrangement [40]. In oxime-modified polypyrrole chemiresistors constructed on cellulose paper, the polypyrrole derivative serves as the primary sensing material, with oxime side chains providing specific recognition sites for organophosphates; optimization of the monomer ratio (pyrrole to oxime-modified pyrrole at 2:8 molar ratio) yielded an increase in electrical conductivity by 10^4^–10^5^ fold upon exposure to three simulants: diethyl cyanophosphonate, diisopropyl fluorophosphate, and diethyl chlorophosphate [41,42].

Flexible configurations have also been explored; for example, a freestanding β-MnO_2_@carbon sheet was developed for detecting the nerve agent simulant DMMP, where the carbon matrix provides a conductive scaffold and the MnO_2_ component contributes catalytic activity for enhanced sensitivity, demonstrating the potential of composite carbon architectures for wearable sensor devices [43].

A representative example of a polymer-based chemiresistive sensor with a well-documented fabrication process is the black phosphorus/carbon nanotube (BP/CNT) composite polypyrrole MEMS device [36]. In this system, polypyrrole serves as the conductive polymer matrix that provides the primary chemiresistive signal transduction pathway, while BP and CNT nanofillers create a hybrid conductive network that enhances charge transport and provides additional adsorption sites for target molecules. The fabrication involves three steps: (i) preparation of BP paste (10 mg BP crystals dispersed in terpineol, sheared at 12,000 r/min for 30 min, and aged for 48 h) and CNT paste (CNTs mixed with terpineol and Triton X-100, ball-milled for 10 h), followed by mixing at a BP:CNT mass ratio of 4:1; (ii) ultrasonic cleaning of the silicon-based MEMS substrate (integrated with platinum heating and interdigitated electrodes) using ethanol and deionized water; and (iii) coating of the composite paste onto the electrode area and vacuum-drying at 150 °C for 120 min. During operation, a voltage of 1.4 V was applied to the heating electrode to accelerate adsorption–desorption equilibrium. The sensor exhibited a linear response to the nerve agent simulant DMMP in the concentration range of 1.5–30 mg/m^3^ (approximately 12.1–242 nM, based on DMMP molecular weight of 124 g/mol), with resistance-change sensitivities of 4.2–13.5% for the pure BP film and 1.8–4.8% for the BP/CNT composite film, response times of 50–75 s, and complete regeneration by N_2_ purging. The superior sensitivity of the pure BP film is attributed to its high carrier mobility and abundant active edge sites, whereas the composite film offers improved mechanical stability and cyclability at the expense of some sensitivity—a trade-off that highlights the importance of balanced material design. This fabrication approach enables batch production of arrayed micro-warning chips for real-time monitoring of organophosphorus vapors in confined spaces [4]. Complementary strategies, such as cobalt phthalocyanine derivative/graphene quantum dot hybrids for room-temperature DMMP (simulant) sensing, further demonstrate the versatility of carbon-based nanohybrids in this field [42].

The electron transfer mechanism offers distinct advantages for practical sensing applications, including direct electrical signal readout, compatibility with miniaturized electronics, and the absence of complex optical components. These features make it particularly suitable for portable and field-deployable devices. However, the mechanism is highly sensitive to environmental conditions: competitive adsorption of water molecules under high humidity, as well as the presence of interfering volatile organic compounds (e.g., ethanol, acetone), can induce non-specific electron transfer and compromise selectivity [38,44,45,46].

In addition to the above complete example, representative material systems based on the electron transfer mechanism are summarised in Table 1, with their detection limits uniformly converted to molar concentration units (nM) to facilitate cross-system comparison.

### 3.2. Hydrogen-Bonding Interactions

As introduced in Section 2.1, hydrogen-bonding interactions enable selective molecular recognition of organophosphorus agents through directional noncovalent binding between hydrogen-bond donors in the polymer (e.g., –OH, –NH_2_, –CONH–) and the P=O group of target analytes. This section reviews representative material systems that exploit this recognition mechanism, emphasizing the structure–selectivity relationships that govern sensor performance and the strategies for integrating hydrogen-bonding recognition with signal transduction platforms [48,49]. As a representative example, Sezigen et al. developed a molecularly imprinted poly-mer sensor based on poly(4-aminobenzoic acid) that recognizes the VX metabolite EMPA through hydrogen bonding, as illustrated in Figure 4.

A representative example is the BHPHFP-modified mesoporous KIT-5 composite QCM sensing material, where BHPHFP serves as the hydrogen-bond acidic molecular recognition layer and KIT-5 provides a high-surface-area porous support to concentrate analyte molecules near the recognition sites. The synthesis involves covalently grafting BHPHFP onto the inner walls of mesoporous KIT-5 silica spheres via a silane coupling agent (3-iodopropyltrimethoxysilane) as a linker, followed by vacuum drying. The resulting functionalized material is coated onto the gold electrode of a quartz crystal microbalance (QCM) to assemble the gas sensor. Upon adsorption into the pores, molecules of the nerve agent simulant DMMP form double hydrogen-bond complexes with the two hydroxyl groups of BHPHFP, and the associated mass change is transduced into a frequency signal. The selectivity of this system arises from the unique electron-withdrawing effect of the hexafluoroisopropylidene moiety in BHPHFP, which enhances the acidity of the hydroxyl groups and strengthens hydrogen-bond formation with the P=O group of organophosphates. This sensor achieves a detection limit for DMMP (simulant) at the ppb level, with minimal signal drift in the presence of ethanol and water vapour, making it suitable for trace organophosphorus screening in complex atmospheric environments [48,50].

Beyond QCM platforms, hydrogen-bonding strategies have also been integrated with conductive nanomaterials (e.g., CNTs, graphene quantum dots) to achieve synergistic improvements, showing broad application prospects in QCM and chemiresistive sensors [36,40]. For instance, polypyrrole-reduced graphene oxide (PPy-rGO) hybrids modified with HFIP groups enable simultaneous hydrogen-bond recognition and charge transfer (Figure 4) [48]. In this hybrid system, polypyrrole serves a dual role: its conductive backbone provides the chemiresistive signal transduction pathway, while the grafted HFIP side chains act as hydrogen-bond donors for selective recognition of organophosphates. The reduced graphene oxide component enhances the overall conductivity and provides additional surface area for analyte adsorption. This dual-mode design effectively suppresses responses to common interferents such as ethanol and acetone while maintaining high sensitivity toward DMMP (nerve agent simulant) [48]. Furthermore, MIPs that exploit hydrogen-bond interactions have been tailored for nerve agent degradation products. A MIP-based electrochemical sensor was developed for the selective detection of the VX metabolite ethyl methylphosphonic acid in human plasma and urine [49], and a MIP-based potentiometric sensor was reported for methylphosphonic acid, a degradation product of CWAs [50].

### 3.3. Fluorescence Quenching

As described in Section 2.1, fluorescence quenching provides a highly sensitive detection mechanism for CWAs through analyte-induced suppression of emission, with the “molecular wire” effect enabling significant signal amplification for conjugated polymers. In this section, we survey representative fluorescence-quenching systems, with emphasis on the polymer’s functional role—as the emissive backbone (conjugated microporous polymers, CMPs), as a host matrix for fluorophores (chitosan nanofibers), or as a structurally engineered platform for sterically controlled emission (boron difluoride complexes). We also highlight how design strategies—including steric regulation, porous structure construction, and MOF–polymer hybridization—are employed to overcome the persistent challenges of solid-state quenching and slow mass transfer [51,52].

Recent developments span a range of polymer architectures. The DAC-TFP conjugated microporous polymer (CMP) exhibits a selective on–off fluorescence response to the nerve agent simulant dimethyl chlorophosphate (DMCP). In this system, the CMP backbone serves as both the emissive fluorophore and the signal-transducing layer; the quenching mechanism involves photoinduced electron transfer (PET) from the electron-rich CMP skeleton to the electron-deficient DMCP molecule, with the conjugated network providing efficient exciton migration pathways for signal amplification [51]. Meanwhile, the three-dimensional cadmium cluster-based coordination polymer NBC-1 enables simultaneous detection of DCP (LOD 32.9 nM) and UO_2_^2+^ (LOD 42.3 nM) via fluorescence quenching, where the coordination polymer framework provides a rigid scaffold that minimizes non-radiative decay pathways and enhances emission stability [51,52]. To address the bottlenecks of solid-state quenching and slow mass transfer, steric engineering strategies have proven effective. By introducing bulky tert-butyl groups into boron difluoride complex films, detrimental π–π stacking is suppressed and a three-dimensional porous network (porosity 38.76%) is constructed to accelerate mass transport. In this system, the boron difluoride complex polymer itself acts as both the fluorescent sensing layer and the structurally engineered host; the tert-butyl substituents serve a dual function—sterically hindering fluorophore aggregation that would otherwise cause self-quenching, while creating permanent free volume that facilitates rapid analyte diffusion to emission sites. This design enabled the BODIQU-tBuCZ sensor to achieve a detection limit as low as 0.001 ppt for DCP vapor (simulant), with a response time of approximately 3 s and stable performance over more than 50 cycling tests—the latter demonstrating excellent reversibility and fatigue resistance [53].

A representative example of a polymer–MOF hybrid ratiometric fluorescent sensor is the UiO-66-NH_2_-supported pyrene-based composite film [54]. In this system, the organic polymer (crosslinked via in-situ thermal polymerization of the pyrene-containing monomer and ethylene glycol dimethacrylate) serves as the fluorescent signal-transducing layer, while the UiO-66-NH_2_ MOF functions dually as a high-surface-area porous support that concentrates analyte molecules and as a catalytic component that promotes analyte hydrolysis for signal generation. The fabrication involves three steps: (i) solvothermal synthesis of UiO-66-NH_2_ from ZrCl_4_ and 2-aminoterephthalic acid in DMF at 120 °C for 24 h; (ii) synthesis of the dual-emission fluorescent monomer via Schiff-base reaction of 4-formylpyrene with ethylenediamine; and (iii) in-situ thermal polymerization at 60 °C within the MOF pores, where the fluorescent monomer and ethylene glycol dimethacrylate crosslinker are fixed via coordination between carboxyl groups and MOF metal nodes. Upon exposure to DCP vapor (simulant), hydrolysis generates H^+^ that protonates the amino groups of the MOF, selectively quenching the short-wavelength pyrene emission while leaving the long-wavelength emission stable. The I_420_/I_510_ ratio exhibits a linear correlation with DCP concentration, enabling ratiometric quantification. This film can be coated onto a portable fluorescence detector window, achieving a response time of less than 5 s for gaseous DCP, making it suitable for on-site ultra-trace rapid quantitative analysis. Notably, UiO-66-NH_2_ possesses enzyme-like activity capable of hydrolyzing DCP to diethyl phosphate and chloride ions, endowing the MOF support with catalytic degradation functionality in addition to its signal transduction role—thus offering a promising material platform for integrated detection–degradation systems [55].

Fluorescence quenching offers exceptional sensitivity (down to ppt level), rapid response times, and compatibility with portable optical devices. However, several practical challenges persist. First, solid-state aggregation-caused quenching (ACQ) remains a major bottleneck for many conventional fluorophores, necessitating steric engineering or porous scaffold incorporation to suppress detrimental π–π stacking. Second, photobleaching of conjugated polymers under continuous UV/Vis excitation limits long-term operational stability, particularly for field-deployable applications requiring extended monitoring periods. Third, the sensitivity of fluorescence to environmental factors—including oxygen (oxidative quenching), humidity, and temperature fluctuations—can compromise measurement reliability in real-world scenarios. Fourth, while fluorescence quenching offers high sensitivity, it generally lacks the structural information provided by ratiometric or time-resolved techniques, making it challenging to distinguish target analytes from interferents that cause non-specific quenching. Future directions focus on: (i) molecular engineering of aggregation-induced emission (AIE) luminogens and sterically protected fluorophores to overcome ACQ; (ii) integration of reference fluorophores for built-in ratiometric calibration; and (iii) development of dual-mode (fluorescence–colorimetric or fluorescence–chemiresistive) sensors to enhance both sensitivity and specificity.

Representative material systems based on fluorescence quenching (ratiometric fluorescence) are summarised in Table 2.

### 3.4. Colorimetric Response

As introduced in Section 2.1, colorimetric detection enables instrument-free, naked-eye identification of CWAs through analyte-induced visible color changes arising from modifications to the polymer’s electronic structure or aggregation state. In this section, we survey representative colorimetric polymer systems, with emphasis on the structure–color relationships that govern visual response—specifically, how molecular-level perturbations (e.g., conformational changes, spirolactam ring-opening, or charge transfer) are transduced into macroscopic color changes. We highlight the functional role of the polymer component in each system, ranging from the conjugated backbone of polydiacetylenes (PDAs) that directly undergoes color transition, to chromophore-grafted polymers that act as signal-amplifying matrices, to supporting polymer frameworks that provide mechanical integrity for practical test strips [51,52].

Polydiacetylene (PDA) and its derivatives remain the most widely studied colorimetric polymer systems. The colorimetric response of PDA arises from a unique topochemical polymerization mechanism: upon UV irradiation, diacetylene monomers undergo 1,4-addition polymerization to form an ene–yne conjugated backbone. The blue-phase PDA features a planar, highly conjugated backbone with a characteristic absorption maximum around 640 nm. Upon external stimuli—such as analyte binding, heat, or mechanical stress—the side-chain packing is disrupted, inducing a conformational change in the main chain that shortens the effective conjugation length, shifting the absorption to ~540 nm and producing a visible blue-to-red transition. This structure–color relationship enables the transduction of molecular recognition events into naked-eye-detectable color changes. The amidoxime-isothiazolone-PDA composite reported by Kim et al. successfully decouples detection and decontamination functions. In this system, the PDA backbone serves as the colorimetric signal transducer, while the amidoxime and isothiazolone side chains act as reactive recognition groups for organophosphorus compounds. Upon exposure, the PDA-oxime matrix undergoes a blue-to-red transition, with characteristic color differences enabling differentiation between real CWAs: the nerve agent GD (soman) induces a red color, while VX appears orange, allowing rapid visual discrimination of these two agents [50]. Beyond PDA, polymers grafted with chromophores such as rhodamine and azobenzene also exhibit excellent colorimetric performance. Upon exposure to the simulants DCP (diethyl chlorophosphate, a nerve agent simulant) or CEES (2-chloroethyl ethyl sulfide, a mustard gas simulant), these systems undergo spirolactam ring opening or intramolecular charge transfer, displaying characteristic colors (e.g., pink, red) with distinct response kinetics that enable differentiation among agent types [49]. Figure 5 illustrates the mechanism and visual test strip photographs of BDHA rhodamine probes for chromo-fluorogenic identification of sarin, tabun and mustard gas simulants [52].

A representative synthesis example is the aldoxime-modified polydiacetylene electrospun nanofiber membrane [52]. In this system, the PDA derivative (PCDA-HBA) serves as the colorimetric active material, with oxime side chains providing specific recognition sites for organophosphates, while poly(arylene ether nitrile) (PEN) functions as a supporting matrix that imparts mechanical integrity and enables electrospinning into a fibrous morphology for enhanced surface area and rapid analyte diffusion. The monomer synthesis involves dissolving 10,12-pentacosadiynoic acid (PCDA) and 2,4-dihydroxybenzaldehyde in dichloromethane with DCC as a condensing agent, stirring for 24 h at room temperature, and purifying the resulting oxime-modified diacetylene monomer (PCDA-HBA) by column chromatography. For membrane fabrication, PCDA-HBA and PEN are co-dissolved in DMF at a 1:5 mass ratio, stirred in the dark for 6 h to obtain the spinning dope, and electrospun at 20 kV with a needle-to-collector distance of 15 cm and a feed rate of 0.001 mm/s. After drying in the dark at room temperature for 48 h, topochemical polymerization is induced by 254 nm UV irradiation, forming a blue conjugated PDA nanofiber membrane. When exposed to real CWAs such as VX or GD vapor, the oxime groups specifically react with the phosphoryl groups, disrupting the side-chain packing and shortening the effective conjugation length of the PDA backbone, which triggers a blue-to-orange-red transition within 1 s. The membrane can be regenerated by immersion in 0.1 mol/L dilute alkali for 10 min, enabling more than 5 repeated detection cycles, and can be integrated into non-woven fabrics for wearable protective warning patches.

The core advantage of colorimetric response polymers is instrument-free detection—results can be read directly by the naked eye without specialized equipment, substantially lowering the detection threshold and operational cost. This feature makes them particularly attractive for field-deployable warning patches and first-responder applications. Furthermore, colorimetric response can be integrated with other signal transduction modes (e.g., fluorescence) to achieve dual-modal detection, enhancing both reliability and information content.

However, several limitations constrain the practical deployment of colorimetric sensors. First, their sensitivity is generally lower than that of fluorescence-based or chemiresistive sensors, making them less suitable for trace-level (sub-ppb) detection. Second, the color change is typically qualitative or semi-quantitative rather than fully quantitative, limiting precise concentration measurement. Third, the stability of the colored state can be affected by ambient light, oxygen, and humidity over extended periods, potentially leading to false-positive or false-negative readings. Fourth, the reversibility of colorimetric responses—particularly for PDA systems where side-chain disruption is involved—is often limited to a finite number of cycles (e.g., ≤5 in the PDA-PEN example above), constraining reusability in continuous monitoring scenarios. Finally, the UV-induced topochemical polymerization required for PDA fabrication adds an additional processing step that may complicate large-scale manufacturing.

Current development trends focus on addressing these limitations through: (i) the design of super-sensitive chromophores with larger molar extinction coefficients to push detection limits toward the ppm/ppb range; (ii) integration with smartphone-based color analysis algorithms for quantitative readout; (iii) encapsulation strategies to protect the colorimetric layer from environmental degradation; and (iv) the construction of multifunctional platforms integrating detection-identification-decontamination, enabling differentiated responses to various toxic agents coupled with simultaneous degradation [57]. Notably, molecular imprinting strategies can also enhance selectivity in other signal transduction modes, as illustrated in Figure 5 for an electrochemical MIP/MWCNT sensor.

## 4. Polymers for Protection & Decontamination

As detailed in Section 2, polymer-based protective materials operate through four primary mechanisms: barrier action, physisorption, filtration, and catalytic degradation. While conventional protective materials—exemplified by butyl rubber—exhibit outstanding barrier performance, this advantage is frequently offset by compromised wearer comfort. In recent years, research efforts have shifted toward developing next-generation polymer composites engineered to integrate high protective efficacy, wear comfort, and self-decontamination functionality through multiscale structural design strategies. This chapter focuses on recent material advances and structure–property relationships, without reiterating basic mechanistic principles.

### 4.1. Barrier

Barrier materials represent the most widely used class of CWA protective systems, with a core trade-off between blocking efficacy and water vapor breathability. Recent advances have optimized this balance via size-exclusion engineering, dense crosslinking, and biomimetic structural design.

Notably, polymers play distinct functional roles across different protective systems, which can be generally categorized into four types: (i) active functional matrix, where polymers themselves serve as the core working components for barrier, adsorption, or molecular recognition; (ii) functionalized substrate, where polymers provide both structural support and participate in interfacial interactions or microenvironment regulation; (iii) structural support/carrier, where polymers only serve as film-forming or fibrous scaffolds to load active components, without contributing core protective activity; (iv) binder/dispersing medium, where polymers are used to disperse and immobilize functional fillers. The functional role of polymers in each system is clarified in the following discussion.

Beyond their specific functional roles, the response of polymer matrices to sorbed toxic substances is also critical to maintaining barrier performance during prolonged exposure. Because chemical agents must first partition into the polymer phase before diffusing through a dense matrix, strong polymer–agent interactions may lead to appreciable sorption and local swelling. The absorbed molecules can increase the intermolecular distance between polymer chains, enlarge the effective free volume, and enhance segmental mobility. Such penetrant-induced plasticization may lower the glass-transition temperature and facilitate subsequent molecular diffusion, potentially resulting in a progressive increase in permeability during prolonged or high-concentration exposure. This phenomenon is particularly relevant to polymers with relatively low cross-linking density or flexible chain architectures, where sufficient conformational freedom exists for chain relaxation after agent uptake. Therefore, the long-term barrier performance of a polymer cannot be evaluated solely from its initial permeability or breakthrough characteristics; resistance to sorption-induced structural relaxation is also critical. Increasing cross-link density is an effective strategy for suppressing this phenomenon because covalent network junctions restrict chain relaxation and limit equilibrium swelling. Rigid chain segments, high crystallinity, and fluorinated groups can provide additional resistance by reducing segmental mobility and/or decreasing the affinity of the polymer surface toward penetrants. For example, highly cross-linked epoxy and fluorinated epoxy coatings have demonstrated enhanced resistance to the CWA simulant DMMP, with increased cross-link density restricting polymer-chain mobility and limiting penetrant-induced transport pathways [58]. Cross-linked fluorinated polyurethane systems also exhibit reduced adsorption and retention of the CWA simulants DMMP and CEES, which has been associated with their higher glass-transition temperature, smaller free-volume pores, reduced microphase separation, and higher fluorine content at the surface [59]. These examples illustrate that combining a rigid or highly cross-linked polymer network with low-surface-energy fluorinated segments can simultaneously reduce penetrant sorption and suppress plasticization, thereby helping to maintain stable barrier performance under prolonged exposure to the corresponding chemical agents or CWA simulants. Among recent developments in barrier materials, cross-linked graphene oxide (GO)/polymer composite films—whose performance rests on size-exclusion principles—stand out as particularly impactful. In a representative study, Kim et al. [60] employed ethylenediamine (EDA) as a cross-linker for GO nanosheets, then combined the cross-linked GO with linear polyethyleneimine (LPEI), which acts as the active barrier matrix and hydrogen-bonding adsorption component, to construct multilayer composite films using spray-assisted layer-by-layer self-assembly. The protective performance of this system emerges from the synergy between size exclusion and hydrogen-bond adsorption. The conformational behavior of LPEI chains is highly sensitive to solution pH: at pH 3 (acidic conditions), the chains adopt an extended conformation, producing a film (designated DPEI/GO) with a well-ordered layered internal structure and a free pore height of just 0.22 nm; at pH 10 (basic conditions), in contrast, the chains collapse into coiled, globular structures, and the resulting film (RPEI/GO) has a disordered internal architecture with a larger free pore height of 0.33 nm. Transmission electron microscopy and X-ray diffraction measurements confirmed that after accounting for the thickness of the GO sheets themselves, the interlayer free space in DPEI/GO is approximately 0.555 nm. This dimension is smaller than the kinetic diameter of DMMP, a widely used nerve agent simulant (0.57–0.60 nm), meaning the film can effectively block DMMP permeation through a strict molecular sieving mechanism. In parallel, amine groups along the LPEI backbone form hydrogen bonds with the P=O groups of DMMP molecules, further slowing the diffusion of agent through the film. Evidence for these hydrogen-bond interactions was obtained via Fourier-transform infrared spectroscopy: following exposure to DMMP vapor, the free N–H stretching peak at 3400 cm^−1^ diminished in intensity, while the hydrogen-bonded N–H peak at 3300 cm^−1^ became more prominent; a concomitant shift in the C=O peak from 1630 cm^−1^ to 1640 cm^−1^ provided additional confirmation. Benefiting from this dual mechanism, the 100 nm-thick DPEI/GO film achieved a DMMP barrier efficiency of 72.65%, with a DMMP vapor permeability of 67.91 g m^−2^ day^−1^—substantially lower than the 248.28 g m^−2^ day^−1^ measured for a bare polyethersulfone (PES) support. At the same time, the film maintained a water vapor permeability of 4524.64 g m^−2^ day^−1^, corresponding to a water/DMMP selectivity of 66.63.

The precision of size-exclusion barrier design has been advanced still further by integrating metal–organic framework (MOF) molecular sieves with the film-forming advantages of GO. In this system, GO serves as a carbon-based film-forming support rather than a polymer matrix, and the size-sieving function originates from ZIF-8 MOF crystals. In work by Kim et al. [61], GO flakes were downsized to nanoscale lateral dimensions (average area 0.013 μm^2^) using a simple ultrasonic treatment. This reduction in flake size markedly increased the density of oxygen-containing functional groups at the sheet edges, creating dense nucleation sites for the growth of ZIF-8 crystals. A continuous, defect-free ZIF-8 layer was subsequently grown in situ on the surface of these small, oxygen-rich GO (SGO) sheets via an impregnation method, yielding a total membrane thickness of around 652 nm, of which approximately 597 nm corresponded to the ZIF-8 layer itself. The barrier action of this composite membrane derives entirely from the well-defined pore structure of ZIF-8: its sodalite-type pores have an aperture of just 3.4 Å, a dimension that falls neatly between the kinetic diameter of water molecules (2.65 Å) and that of DMMP (5.71 Å). This exquisitely precise sieving effect reduced the DMMP permeability of the ZIF-8/SGO membrane to 10.8 g m^−2^ day^−1^, representing a 97% decrease relative to the 330.5 g m^−2^ day^−1^ of the plain SGO membrane. Water vapor permeability, meanwhile, remained at 3373 g m^−2^ day^−1^, delivering an exceptionally high water/DMMP selectivity of 312—superior to the vast majority of selective permeation membranes reported in the literature at that time. This study clearly illustrates that combining the precise molecular sieving of MOF ultramicropores with the processable, film-forming characteristics of GO offers a viable route to high-performance barrier materials against CWA simulants while preserving breathability, although validation against actual CWAs remains necessary.

Size exclusion, whether achieved through nanoscale interlayer gaps or the well-defined pore systems of MOFs, represents one major design paradigm; a second, equally important approach is to construct dense polymer barriers by tailoring molecular-scale cross-linking networks. In one representative study, Zhang et al. [62] prepared a fluorosilane-epoxyacrylate ternary prepolymer (PAK), in which the polymer itself serves as the sole active barrier component, functionalized with epoxy groups, fluoroalkyl chains, and siloxane side groups via radical polymerization, then cured and cross-linked the material using a mixed curing agent system comprising 4,4′-diaminodicyclohexylmethane (DDCM) and fluorosiloxane. The barrier action of the resulting coating stems from the interplay of several physicochemical factors. Dynamic mechanical characterization demonstrated that lengthening the fluorosiloxane curing agent chains increased cross-linking density from 1877 to 3921 mol·m^−3^. This high cross-linking density strongly constrains polymer chain mobility, which in turn lowers the diffusivity of agent molecules within the coating. At the same time, positron annihilation lifetime spectroscopy measurements showed that incorporation of long-chain fluorosiloxanes increased the average free-volume pore size from 123.83 to 215.16 Å^3^. Despite this enlargement, the relative free-volume fraction (FFV_r_) did not rise correspondingly, and DMMP permeability remained effectively suppressed—a consequence of both the higher cross-linking density and the weakly repulsive interactions between fluorinated side chains and DMMP. A further key effect is the surface migration of fluorinated segments during curing, which, together with micro- and nanoscale surface roughness arising from microphase separation and solvent evaporation, yields a low-surface-energy surface with both superhydrophobic and superoleophobic characteristics. The coating displays contact angles of 120° against DMMP and 109° against the corrosive simulant CEES, and delivers barrier efficiencies of 99.96% and 100%, respectively, for the two agents. These findings highlight how bulk-phase dense cross-linking to suppress diffusion and low-energy rough surfaces to repel wetting can act in concert to deliver strong barrier protection against the corresponding CWA simulants.

Whereas the designs discussed above are based on dense structures or molecular sieving, biomimetic structural engineering offers a distinct strategy for reconciling high elasticity with high barrier performance. Song et al. [63] drew inspiration from the natural extracellular matrix (ECM) to construct a bicomponent biomimetic barrier layer with a “fibrous scaffold-elastic matrix” architecture. First, an electrospun thermoplastic polyurethane (TPU)/fluorinated polyurethane (FPU) nanofiber membrane was prepared to serve as the elastic structural scaffold polymer; this was then infiltrated under vacuum with an elastic matrix consisting of styrene–butadiene–styrene block copolymer (SBS), which acts as the polymer binder and adsorption matrix, and activated carbon as the active adsorbent component. Unlike conventional barrier layers, the system does not function exclusively through physical interception. The elastic fibrous scaffold provides mechanical support and outstanding resilience, recovering fully from 80% strain and retaining structural integrity after 1000 stretching cycles. Meanwhile, the SBS/activated carbon matrix filling the interstitial spaces between fibers captures agent molecules through physical adsorption via the extensive microporosity of the activated carbon. Together, these two components endow the composite fabric with a CEES permeability of just 0.00214 μg cm^−1^ min^−1^, substantially below that of commercial Tychem protective materials. Most importantly, barrier performance showed no measurable decline after 100 cycles of 50% tensile strain, because the fiber framework and elastic matrix deform in a coordinated manner under strain rather than separating at their interface. This ECM-mimetic design concept thus addresses a longstanding limitation of conventional barrier materials—the loss of protective performance under dynamic use due to structural failure.

Building on such nanofiber-based concepts, researchers have further expanded barrier protection applications by combining nanofiber physical interception with an ultrathin hydrophobic coating. Li et al. [64] developed a composite membrane featuring both high breathability and durable water repellency, fabricated by electrospinning a polyimide (PI) nanofiber membrane, which serves as the fibrous structural support polymer, followed by chemical vapor deposition of an ultrathin polydimethylsiloxane (PDMS) layer as the surface-functionalized polymer barrier. Protection in this system arises from two complementary mechanisms: physical capture of aerosol particles by the three-dimensional PI nanofiber network, and hydrophobic barrier action provided by the PDMS coating. By tuning the polyacrylamide concentration (12 wt%) and solvent composition (DMF/acetone = 6:4) in the spinning dope, the authors obtained uniform, bead-free PI nanofibers with an average diameter of 500 nm and a tensile strength of 13.12 MPa, markedly exceeding the performance of most electrospun nanofiber membranes. The optimized membrane exhibited filtration efficiencies of 96.13% for 300 nm NaCl aerosols, 99.42% for 20 nm ultrafine particles, and 99.48% for Staphylococcus aureus aerosols, while sustaining an air permeability of 265.44 mm s^−1^—40 to 160 times higher than typical commercial protective fabric materials. Applied via chemical vapor deposition, the ultrathin PDMS layer endows the membrane with excellent hydrophobicity—evidenced by a water contact angle above 135°—as well as improved flame resistance, all without compromising filtration performance or air permeability. When exposed directly to flame, the PDMS-functionalized membrane shows virtually no sustained combustion; by comparison, neat PI membranes, although classified as self-extinguishing, still exhibit partial burning behavior. A further key advantage is its operational durability: after five repeated disinfection cycles using alcohol, chlorine-based disinfectants, and UV irradiation, neither the filtration efficiency nor the hydrophobicity of the composite membrane decreased significantly. This stability underscores the considerable promise of the material for reusable protective clothing applications.

Taken together, the barrier protection materials reviewed above fall into four core design strategies: molecular sieving, which blocks agent molecules via interlayer voids (0.22 nm) or intrinsic MOF metal–organic framework channels (3.4 Å) and yields a water/DMMP selectivity as high as 312; dense cross-linking, which suppresses agent diffusion by restricting polymer segmental motion at cross-linking densities up to 3921 mol·m^−3^ and, paired with a superhydrophobic–superoleophobic surface, achieves a barrier efficiency above 99.9% against the tested CWA simulants; fibrous network interception, which captures aerosol particles through 500 nm-diameter nanofiber networks and delivers an air permeability as high as 265.44 mm s^−1^; and interception–adsorption synergy, in which an adsorptive matrix is embedded within an elastic framework to preserve stable barrier performance even under dynamic stretching. A unifying trend across all four approaches is the evolution from single-mechanism barrier action to multi-mechanism synergistic design, with size-exclusion configurations offering the optimal balance between retention performance and breathability.

In summary, polymers in barrier systems mostly serve as active barrier matrices or structural supports, with size-exclusion fillers such as GO and MOFs incorporated to enhance blocking performance.

From the perspective of scalable manufacturing and cost, dense cross-linked polymer coatings and electrospun nanofiber membranes have relatively favorable industrialization foundations. Fluorosilicone-epoxy acrylate coatings can be fabricated via conventional roll-coating or spray-coating processes, using industrially mass-produced raw materials with controllable costs, which can be readily scaled up to continuous production lines. For electrospun nanofiber membranes, pilot-scale continuous production has been achieved; matrix resins such as PI and TPU are low-cost and the fabrication process is technically mature. In contrast, scalable production of GO/MOF composite membranes and MOF molecular sieve membranes still faces challenges. The controlled synthesis of nanoscale GO and the uniform in-situ growth of MOF crystals on substrate surfaces are highly sensitive to processing conditions, resulting in unsatisfactory batch-to-batch reproducibility. Moreover, the high cost of MOF ligands and GO raw materials raises concerns regarding the economic viability of large-scale applications. Overall, while the performance of barrier materials has been extensively investigated, the development of low-cost, mass-producible fabrication technologies for practical deployment remains a key direction for future breakthroughs.

Key structural and performance metrics of representative materials are compiled in Table 3.

### 4.2. Physical Adsorption

Protection based on physical adsorption operates chiefly through three mechanisms: van der Waals forces, capillary coalescence, and specific noncovalent interactions, all of which are supported by the high specific surface area characteristic of porous polymers. Unlike barrier materials, which depend on dense structures to block permeation, adsorptive materials sequester toxic molecules via an interconnected, open porous network. This open structure means that high adsorption capacity can be achieved without sacrificing breathability, provided that pore structure and adsorption sites are designed synergistically.

Physisorptive materials capture CWA molecules or representative CWA simulants via noncovalent interactions within porous polymer networks, offering high adsorption capacity without compromising breathability. Representative porous polymer systems and their adsorption performance are summarized below.

PIMs, in which the polymer itself acts as the active adsorption material, are a particularly attractive class of physisorptive materials due to their rigid molecular backbones and permanent intrinsic microporosity. In one study, Wang et al. [34] used electrospinning to fabricate microfiber membranes from PIM-1, in which adsorption is driven primarily by capillary coalescence within the high-surface-area pore network. PIM-1 possesses a native hierarchical pore structure that gives it a Brunauer–Emmett–Teller (BET) specific surface area as high as 650 m^2^ g^−1^; its abundant micro- and mesopores enable strong trapping of DMMP molecules through cumulative van der Waals interactions. The resulting PIM-1 fiber membrane reached a DMMP adsorption capacity of 47 mg g^−1^, approximately double the 25 mg g^−1^ measured for commercial activated carbon fabric. This result underscores how high specific surface area and continuous, interconnected pores are decisive factors in maximizing physical adsorption performance. That said, unmodified PIM-1 relies almost entirely on nonspecific van der Waals interactions, and its selectivity toward DMMP remains limited.

A second class of materials showing promise for selective physical adsorption of organophosphorus compounds is coordination polymers (CPs), where the polymer serves as the active adsorption and recognition material, combining accessible Lewis acid sites with rich hydrogen-bonding functionality. Abuzalat et al. [30] developed a novel zirconium-based CP, designated Zr-(OC-AMAM-CO)CP, constructed from Zr(IV) oxo clusters coordinated to an amide-functionalized dicarboxylic acid ligand. X-ray photoelectron spectroscopy (XPS) analysis indicates that Zr(IV) ions bind preferentially to the terminal carboxylate oxygen atoms of the ligand, leaving the amide groups uncoordinated and available to act as hydrogen-bonding sites for molecular recognition. When exposed to DMMP under visible light, the material undergoes a rapid, reversible color change from orange-yellow to dark green in less than 4 s. Reflectance spectroscopy shows that this response corresponds to a sharp drop in reflectance at 507 nm, along with a blue shift in the absorption peak to 465 nm. Mechanistically, selective adsorption is mediated by hydrogen bonding: the P=O group of DMMP acts as a hydrogen-bond acceptor, forming P=O⋯H-O interactions with bridging hydroxyl groups in the Zr-CP framework. The high selectivity of this interaction was confirmed via competitive adsorption experiments, in which common interferents such as water, ethanol, and CEES induced only small changes in reflectance and no peak shift. An additional notable feature is that ultraviolet excitation at 365 nm triggers the formation of direct P=O-Zr coordination bonds, switching the adsorption mode from reversible physical adsorption to irreversible chemisorption and producing a fluorescence enhancement effect. This ability to toggle between physical and chemical adsorption within a single material system provides a useful strategy for developing multimodal sensing materials that combine fast initial recognition with amplified signal output.

Adsorption performance can be further improved by functionalizing porous polymers with targeted affinity groups. Jung et al. [36] demonstrated this approach through post-synthetic modification of the PIM-1 polymer matrix with xylamine oxime groups, yielding the functionalized polymer PIM-1-AX as the active adsorption component. the improved adsorption arises from a multipoint interaction mechanism: the nitrogen and oxygen atoms in the xylamine oxime moieties serve as both hydrogen-bond donors and acceptors, forming multiple hydrogen bonds and coordination interactions with organophosphorus molecules and thus substantially increasing binding affinity. In pH 8.06 borate buffer, PIM-1-AX was able to adsorb and simultaneously degrade 84% of DMNP over 48 h. Activating the material with supercritical CO_2_ further increased its specific surface area, boosting the overall adsorption–hydrolysis rate by 1.7-fold. Although PIM-1-AX exhibits catalytic behavior, its central advantage lies in the strong enhancement of physical adsorption affinity achieved through targeted functionalization. This work illustrates a broader trend in the field: a move away from nonspecific, van der Waals-driven adsorption toward functional group-mediated, synergistic adsorption systems with higher selectivity and capacity.

In summary, polymers in physisorption systems generally act as the active adsorption and recognition components, with tailored porous structures and functional groups directly determining adsorption capacity and selectivity. It should be noted that the representative adsorption data discussed above are predominantly obtained using CWA simulants such as DMMP and DMNP; therefore, these results demonstrate the adsorption capability of the corresponding model compounds rather than direct validation against actual CWAs.

Regarding scalable production and cost, PIM-based materials involve relatively complex synthetic procedures. The monomer preparation and polymerization processes are technically challenging, and the raw material cost is significantly higher than that of conventional porous materials. Currently, PIMs are mainly synthesized in small batches at the laboratory scale, and mature mass-production routes have not been established. In comparison, traditional adsorbent materials such as activated carbon and porous resins feature low cost and sufficient production capacity, but suffer from limited adsorption selectivity and capacity. Coordination polymers exhibit excellent selectivity, but the high cost of metal ligands and the difficulty in controlling uniformity during batch synthesis hinder their large-scale application. The development of low-cost, scalable synthetic routes for porous polymers is a critical step to promote the practical application of physisorption-based protective materials.

### 4.3. Filtration

Filtration and interception strategies act primarily on aerosolized chemical-agent-containing particulates, relying on four distinct capture mechanisms: inertial impaction, direct interception, Brownian diffusive deposition, and electrostatic adsorption. Among available filter materials, electrospun polymer nanofiber membranes stand out for their ability to deliver both high filtration efficiency and low breathing resistance, a performance profile enabled by their fine fiber diameters, tightly controlled pore sizes, and high overall porosity. Beyond filtration performance, these structural characteristics also make nanofiber membranes exceptionally well-suited as versatile platforms for building multifunctional protective systems.

One major line of development in modern filtration materials has been to merge basic physical filtration with functionalized interception mechanisms. In one representative study, Wang et al. [34] prepared a PIM/PAN heterostructured composite membrane using sequential layer-by-layer electrospinning, with a polyacrylonitrile (PAN) nanofiber layer as the structural reinforcing polymer scaffold and a PIM-1 microfiber layer as the functional adsorption polymer (Figure 6). Filtration in this system proceeds through two synergistic pathways. First, the densely assembled PAN nanofibers create a three-dimensional network with small, uniform pores that trap aerosol particles efficiently via inertial impaction and direct interception. Second, the highly polar nitrile groups along the PAN backbone generate strong dipole–dipole interactions between adjacent fibers, which markedly improves the mechanical robustness of the membrane and prevents deformation or damage to the fiber network during service. The resulting composite membrane achieves PM_2.5_ and PM_10_ filtration efficiencies of 99.75% and 99.87%, respectively—performance comparable to commercial polypropylene melt-blown fabrics—while boosting tensile strength from 0.01 MPa for neat PIM-1 to 0.78 MPa.

Beyond mechanical reinforcement, the introduction of functional fillers into electrospun fiber networks provides an alternative route to simultaneously enhance air permeability and sorption capacity. Lee et al. [65] incorporated micrometer-sized activated carbon (AC, 5–20 µm) and copper(II) oxide (CuO) nanoparticles into electrospun PAN nanofibers (≈200 nm diameter), where the polymer acts as the fibrous support carrier. Unlike conventional additives that densify the fiber matrix, the coarse AC granules physically disrupt fiber packing, creating enlarged inter-fiber spacing that significantly improves air permeability. Simultaneously, the AC particles serve as high-capacity adsorbents for gaseous CWAs via their intrinsic microporosity (BET surface area ≈ 2000 m^2^ g^−1^), while CuO nanoparticles provide additional degradation activity toward nerve agents (e.g., soman) through surface-mediated hydrolysis. The optimized formulation—8% PAN with 10 wt% AC and 3 wt% CuO—achieved a sixfold increase in air permeability relative to neat PAN membranes, while maintaining ≈94% aerosol filtration efficiency against 100 nm polystyrene particles (size analogous to SARS-CoV-2). The Swatch test further confirmed that this membrane reduced cumulative penetration of the actual nerve agent GD by 20-fold compared to control samples over a 3 h exposure period, establishing it as a promising candidate for protective suits requiring both breathability and broad-spectrum chemical/biological protection.

A more transformative advance in next-generation filtration materials is the addition of catalytic self-detoxification capability to existing physical filtration structures. Qiu et al. [66] demonstrated this approach by growing zirconium hydroxide (Zr(OH)_4_) nanoparticles directly on the surface of PAN nanofibers, producing a Zr(OH)_4_ @PAN composite nanofiber membrane with dual filtration and degradation functionality. The system operates via two complementary physicochemical modes. The PAN fiber network provides the primary physical barrier, capturing toxic aerosols with an interception efficiency of up to 99.945%. At the same time, Zr(OH)_4_ nanoparticles anchored to the fiber surfaces function as Lewis acid catalytic sites. The unsaturated coordination sites on Zr interact with the sulfur atoms of CEES molecules, polarizing the S-C bond and promoting selective oxidation of the highly toxic sulfide into its less toxic sulfoxide derivative. This reaction proceeds under ambient temperature and humidity, without requiring external oxidants or buffer solutions, and reaches a degradation efficiency of over 90% within 4 h. By combining the rapid, immediate capture of physical interception with the permanent, thorough detoxification of chemical degradation in a single fibrous membrane, this work achieves a fully integrated filtration and self-detoxification system.

In summary, polymers in filtration systems mainly function as fibrous structural carriers, providing a porous network backbone while functional fillers are introduced to achieve additional adsorption or degradation capabilities.

From the perspective of industrialization potential, the scalable manufacturing technology for electrospun nanofiber filtration membranes is relatively mature. Matrix resins such as PAN are low-cost, and continuous electrospinning production lines enable large-area, high-efficiency manufacturing with controllable overall cost, creating conditions for rapid practical deployment. However, functional modifications (such as in-situ MOF growth and metal oxide nanoparticle loading) increase process complexity and raw material costs. Furthermore, technical challenges remain in achieving uniform particle loading and robust interfacial adhesion during scale-up production. Further process optimization is required to balance performance and economic efficiency.

### 4.4. Catalytic Degradation

Catalytic degradation enables detoxification of CWAs and their representative simulants via two primary pathways: hydrolysis of P-X bonds in nerve agents and oxidation of thioether moieties in blister agents. A central barrier to real-world deployment is sustaining the efficiency and stability of catalytic active sites under solid-state conditions—without external buffers or added liquid water—across a broad humidity range.

Because most representative catalytic studies discussed below employ simulants such as DMNP and CEES rather than actual CWAs, the reported conversion rates and half-lives are interpreted as performance against the experimentally tested compounds. Direct extrapolation to actual CWAs requires further validation under comparable conditions.

Zr-based metal–organic frameworks (Zr-MOFs) have emerged as benchmark catalysts for nerve agent hydrolysis owing to the Lewis acidity of their Zr_6_ oxo clusters, yet their powdered form and reliance on exogenous alkaline buffers severely limit practical application. One widely explored solution is the construction of in situ catalytic microenvironments by incorporating basic or amphoteric molecules into MOF pore networks. Notably, the following two MOF modification studies involve no polymer components; they are presented as performance benchmarks for comparison with polymer-supported catalytic systems. Wu et al. [67] introduced alkanolamines (e.g., monoethanolamine (MEA)) into MOF-808 channels, where amine moieties function as solid bases to promote water dissociation into nucleophilic OH^-^ species and hydroxyl groups enhance hydrophilicity to trap atmospheric moisture. This modification boosted DMNP conversion from 31.0% to 82.1% after 1 h at 60% relative humidity (RH), and achieved near-complete conversion at 80% RH. Jiang et al. [68] embedded amphoteric dopamine (DA) into UiO-66-NH_2_; the synergistic buffering effect of catechol and amino groups reduced the DMNP hydrolysis half-life in pure water from 22.4 to 7.0 min. When applied to MOF-808, this strategy further shortened the half-life to 0.64 min, ranking among the fastest hydrolysis rates reported for pure-water systems. Luo et al. [69] fabricated a mixed matrix membrane by dispersing MOF-808 in a PVDF/PVP/imidazole polymer blend, where polymers act as the film-forming matrix and microenvironment regulator. In this system, PVP serves as a moisture sorbent and imidazole provides basic sites, enabling solid-state hydrolysis with a t_1_/_2_ of 5 min at 98% RH. This work marks the first demonstration of solid-state catalysis in a MOF/polymer composite membrane without exogenous liquid water. Li et al. [70] grew MOF-808 in situ on FeCoO_x_@PAN electrospun fibers and grafted 4,5-imidazoledicarboxylic acid (IMDC) onto the framework. density functional theory (DFT) calculations show that IMDC functionalization lowers the hydrolysis energy barrier from 1.47 to 0.92 eV (a 37% reduction), cutting the DMNP half-life to 6.6 s and enabling full conversion within 1 min—the fastest reaction rate reported to date for MOF/polymer composite catalysts.

Beyond microenvironment engineering, tuning the electronic structure of catalytic active sites can deliver substantial improvements in intrinsic activity. Cai et al. [71] anchored isolated Cu single atoms onto the amino sites of UiO-66-NH_2_ via Cu–N_2_ coordination, shifting the primary catalytic center from Zr_6_ clusters to Cu single atoms. XPS characterization and DFT calculations indicate that the organophosphorus substrate binds to Cu sites in a monodentate configuration via the P=O moiety; the resulting charge redistribution weakens the P=O bond and markedly reduces the hydrolysis energy barrier, giving a t_1_/_2_ of ~2 min and an ~8-fold increase in initial hydrolysis rate. Alongside activity enhancement, shaping powdered MOFs into recyclable macroscopic forms is critical for practical implementation. Kiaei et al. [72] encapsulated UiO-66-NH_2_ within a PES matrix where the polymer acts as the shaping binder and structural support, via phase inversion to produce millimeter-sized spherical composite beads, which retained a BET specific surface area of 365–560 m^2^ g^−1^ and exhibited a DMNP hydrolysis half-life of 5.09 min with no loss of activity over three reaction cycles. Yan et al. [73] grew MOF-808 and UiO-66-NH_2_ sequentially on sulfonated PI nanofibers, where the polymer serves as the fibrous support carrier, achieving a MOF loading of 65.2 wt%. The dual-MOF synergy enabled 99.5% DMNP removal within 20 min (t_1_/_2_ = 2.73 min) and 99.6% CEES removal within 24 h, with >96% of initial activity retained after five cycles. Wu et al. [74] further developed PAN@Zr(OH)_4_@MOF-808 core–shell nanofibers, in which the outer MOF-808 layer mediates rapid DMNP hydrolysis (t_1_/_2_ = 1.19 min) and the inner Zr(OH)_4_ layer catalyzes CEES degradation. The MOF shell also acts as an adsorption barrier to slow inward CEES diffusion, establishing a sequential “adsorption–controlled release–catalysis” mechanism. Under dual-simulant coexistence conditions, complete DMNP conversion was achieved within 2 h, with CEES penetration remaining below 1%.

Three-dimensional scaffold integration represents another design direction that addresses both mass transfer and structural stability requirements for device-level applications. Niu et al. [12] employed graphene oxide aerogels (GAs) as a multifunctional scaffold to integrate UiO-66-NH_2_@DA MOF and MIPs as the molecularly imprinted polymer protective coating layer. Quantitative tuning of carboxyl groups on the GA backbone enables self-buffering behavior, maintaining pure water at pH 9–11 without external additives. The aerogel’s three-dimensional hierarchical porous structure accelerates substrate mass transfer, while the MIP coating acts as a protective layer, reducing Zr leaching from 2.65% to 0.04%. In pure water, this ternary architecture achieves a DMNP hydrolysis rate constant of 0.2227 min^−1^, corresponding to a half-life of 3.11 min. It retains over 93.5% of its initial activity after ten consecutive cycles, and can be fabricated into continuous-flow filtration modules to enable complete degradation under dynamic operating conditions.

Collectively, these developments trace a clear evolutionary trajectory for catalytic degradation materials: the field is shifting from buffer-dependent powder catalysts toward self-sufficient systems characterized by inherent buffering capacity, wide environmental adaptability, and ease of integration. Functionalization with alkanolamines and amphoteric guest molecules has eliminated reliance on externally supplied buffers, whereas single-atom catalytic designs have pushed beyond the intrinsic activity limits of conventional Zr_6_ cluster sites. Macroscopic shaping strategies—including phase-inversion composite beads, core–shell nanofibers, and monolithic aerogels—have bridged the gap between laboratory synthesis and practical deployment in protective equipment.

In summary, polymers in catalytic degradation systems mostly serve as structural supports, film-forming matrices, or protective coatings, while the core catalytic activity originates from MOFs, single-atom catalysts, or metal oxides. This division clarifies that polymer matrices enable the practical deployment of high-performance catalytic components through processable shaping and integration.

Scalable manufacturing and cost represent core constraints for the practical deployment of catalytic degradation materials. For Zr-MOFs, the ligand synthesis and solvothermal preparation processes are complex, and zirconium salts are relatively expensive. Large-scale production of pure MOF powders is technically challenging and costly. Single-atom catalysts require even more elaborate fabrication with stringent control over synthesis conditions, and are currently only producible at the laboratory scale at extremely high cost. In contrast, the composite strategy of immobilizing MOFs and catalytic components on polymer matrices or fiber surfaces can substantially reduce the consumption of noble metals and MOFs, thereby lowering the overall cost. However, the scale-up effects of processes such as in-situ growth and functional grafting remain unclear, and batch-to-batch stability and long-term cycling durability still require pilot-scale verification. Overall, while significant breakthroughs have been achieved in the performance of catalytic degradation materials, the development of low-cost, scalable, and controllable fabrication processes remains a key challenge for future industrialization.

Across the protection and decontamination studies reviewed in this chapter, the level of experimental evidence varies substantially. Results obtained using actual CWAs provide direct evidence of agent-specific protective or decontamination performance, whereas studies based on simulants such as DMMP, DMNP, DCP, DMCP, and CEES primarily establish proof-of-concept relationships between material structure and chemical-agent-related behavior. Although these simulants are valuable for safe and systematic materials screening, differences in molecular size, volatility, reactivity, and physicochemical properties may affect their interactions with polymer matrices and functional components. Therefore, simulant-based results should be interpreted as evidence of potential applicability rather than as direct equivalence to actual-CWA performance.

To facilitate systematic comparison of the underlying mechanisms and performance boundaries across different systems, key structural and catalytic parameters of representative materials are compiled in Table 4.

## 5. Perspective and Conclusions

### 5.1. Conclusions

This review has systematically surveyed recent advances in polymer-based materials for the detection and protection against CWAs, with particular emphasis on the underlying interaction mechanisms and the structure–property relationships that govern material performance.

For CWAs detection, polymer-based sensing systems operate through four principal mechanisms—electron transfer, hydrogen-bonding interactions, fluorescence quenching, and colorimetric response—each offering distinct advantages for specific application scenarios. Conductive polymers and their composites provide direct electrical signal transduction amenable to miniaturization and integration; hydrogen-bond acidic polymers enable highly selective recognition of organophosphorus agents through specific noncovalent interactions; conjugated polymers exploit the “molecular wire” effect to achieve ultrahigh sensitivity via fluorescence quenching; and polydiacetylene-based systems allow instrument-free, naked-eye detection through visible color changes. The synergistic integration of multiple sensing mechanisms within a single material system has emerged as a particularly promising strategy for enhancing sensitivity, selectivity, and anti-interference capability.

For CWAs protection, current polymer-based materials operate through four complementary strategies: barrier action, physical adsorption, filtration, and catalytic degradation. Barrier materials—including cross-linked dense networks, graphene-based laminates, and MOF-integrated membranes—suppress agent permeation through size exclusion or suppressed diffusion while maintaining breathability. Porous polymers such as PIMs and coordination polymers provide high-capacity adsorption through tailored surface functionality and specific noncovalent interactions. Electrospun nanofiber membranes effectively capture aerosolized agents with low air resistance, offering an excellent platform for integrating multiple protective functions. Catalytic composites incorporating Zr-MOFs, single-atom catalysts, or functionalized polymers enable the hydrolysis of nerve agents and oxidation of blister agents under ambient conditions, with recent advances achieving self-buffering and solid-state operation that eliminate reliance on exogenous additives.

A clear evolutionary trend is evident across both detection and protection domains: the field is shifting from single-function, passive materials toward multifunctional, active systems that combine recognition, protection, and self-detoxification capabilities within a unified polymer platform.

### 5.2. Future Perspectives

Despite the significant progress reviewed herein, several critical challenges remain to be addressed before polymer-based CWAs detection and protection technologies can achieve widespread practical deployment.

Selectivity and environmental tolerance. For sensing applications, the concurrent presence of humidity, organic volatiles, and other interferents in real-world environments continues to compromise sensor selectivity. While hydrogen-bond acidic polymers offer inherent specificity toward organophosphorus compounds, further improvement in anti-interference capability is required. For protective materials, maintaining high barrier or catalytic performance across broad ranges of temperature and humidity remains a substantial challenge, particularly for solid-state catalytic systems that currently show strong humidity dependence.

Balancing protection and wearer comfort. The trade-off between barrier performance and breathability persists as a central challenge in protective materials. Although molecular sieving membranes and electrospun nanofiber filters have made notable progress in reconciling these competing demands, achieving truly comfortable protective clothing that simultaneously offers high protection against both vapor-phase and aerosolized CWAs remains an elusive goal.

Scalability and long-term stability. Many of the high-performance materials discussed in this review—particularly MOF/polymer composites and single-atom catalysts—have been demonstrated primarily at the laboratory scale. Translating these materials into industrially viable products requires addressing issues of batch-to-batch reproducibility, mechanical robustness, and long-term storage stability. The development of scalable manufacturing processes, such as continuous electrospinning and roll-to-roll coating, will be essential for practical implementation.

Integration of multifunctionality. The next frontier lies in the seamless integration of detection, protection, and decontamination functions into a single material system. Emerging concepts such as wearable sensor arrays that provide real-time threat warning, self-detoxifying fabrics that eliminate toxic agents upon contact, and smart materials that adapt their protective performance in response to environmental stimuli represent exciting directions that are beginning to move from concept toward reality.

Computational design and data-driven discovery. The vast chemical space available for polymer and composite design calls for accelerated discovery approaches. Machine learning and high-throughput computational screening hold considerable promise for predicting polymer–CWA interaction energies, optimizing pore architectures, and identifying promising catalytic sites, thereby reducing the reliance on trial-and-error experimentation.

Looking ahead, we anticipate that the convergence of polymer chemistry, nanotechnology, and materials engineering will continue to drive innovation in this critical field. The development of next-generation chemical defense materials will likely be characterized by intelligent, responsive systems that not only protect against CWAs but also actively sense and neutralize them, ultimately providing comprehensive and reliable protection for military personnel, first responders, and civilian populations against the persistent threat of CWAs.

## Figures and Tables

**Figure 1:**
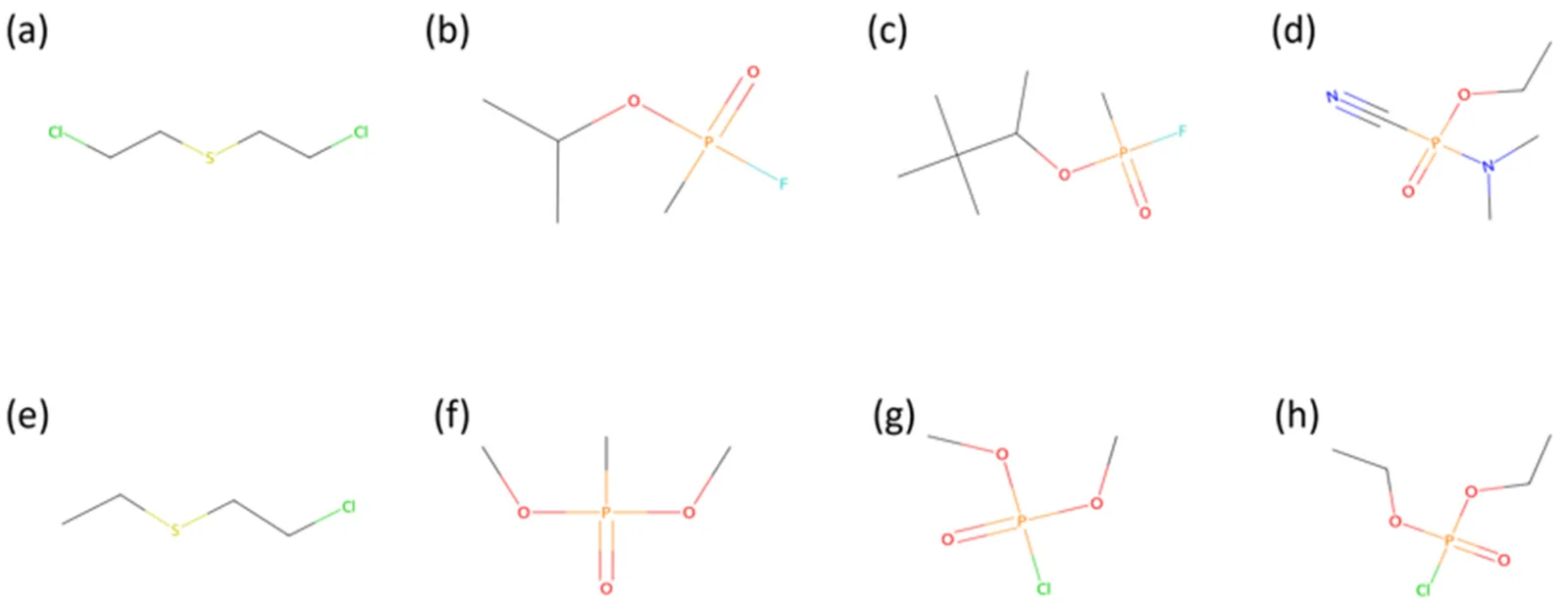
Representative chemical structures of major CWAs and their corresponding simulants discussed in this review: (**a**) Sulfur mustard (HD); (**b**) Sarin (GB); (**c**) Soman (GD); (**d**) Tabun (GA); (**e**) 2-CEES; (**f**) DMMP; (**g**) DMCP; (**h**) DCP.

**Figure 2:**
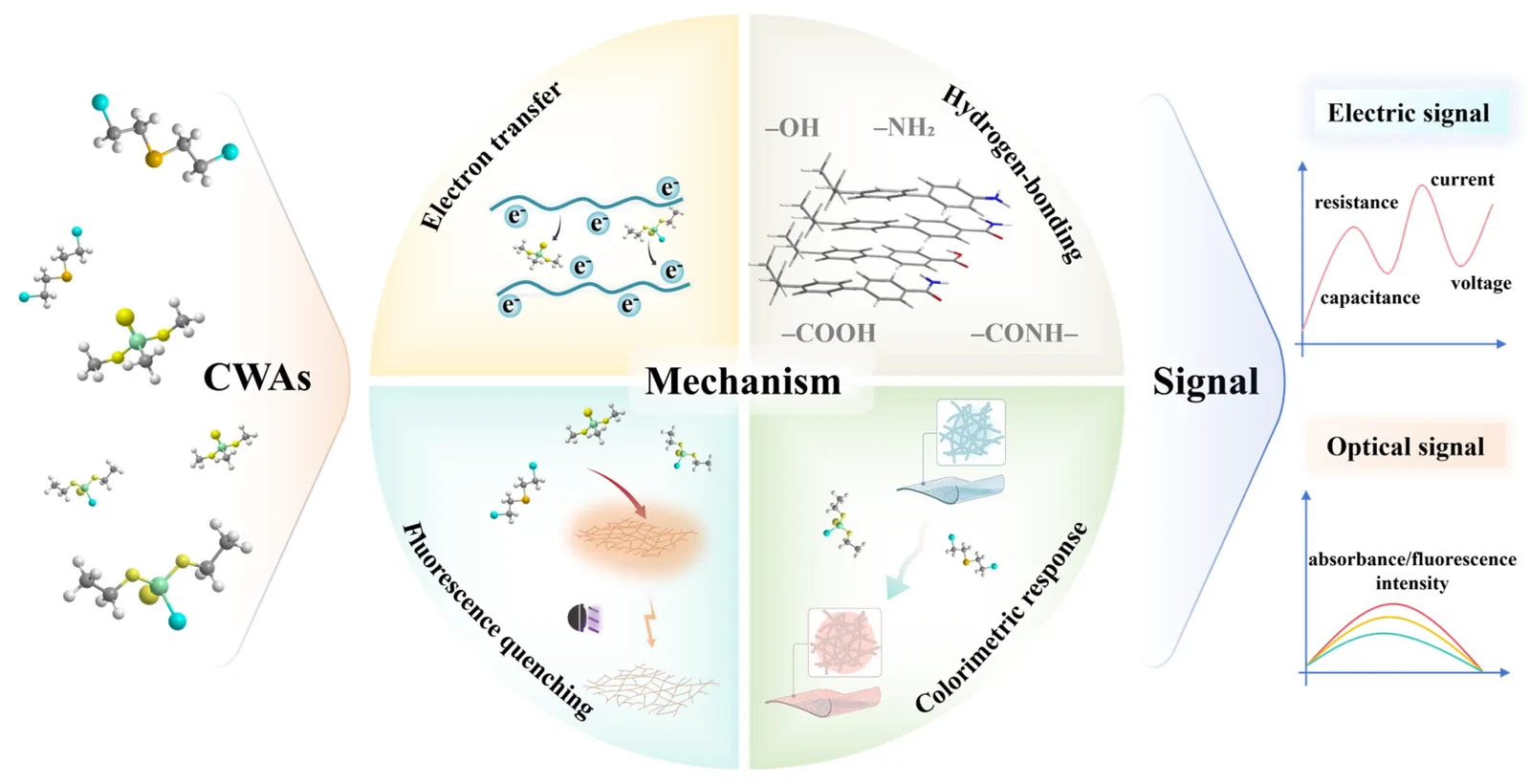
Mechanisms of CWAs Sensing by Polymers, including electron transfer, hydrogen bonding, fluo-rescence quenching, and colorimetric response, and their conversion into electrical or optical signals. The arrows indicate the direction of the interaction-induced sensing processes and subsequent signal transduction.

**Figure 3:**
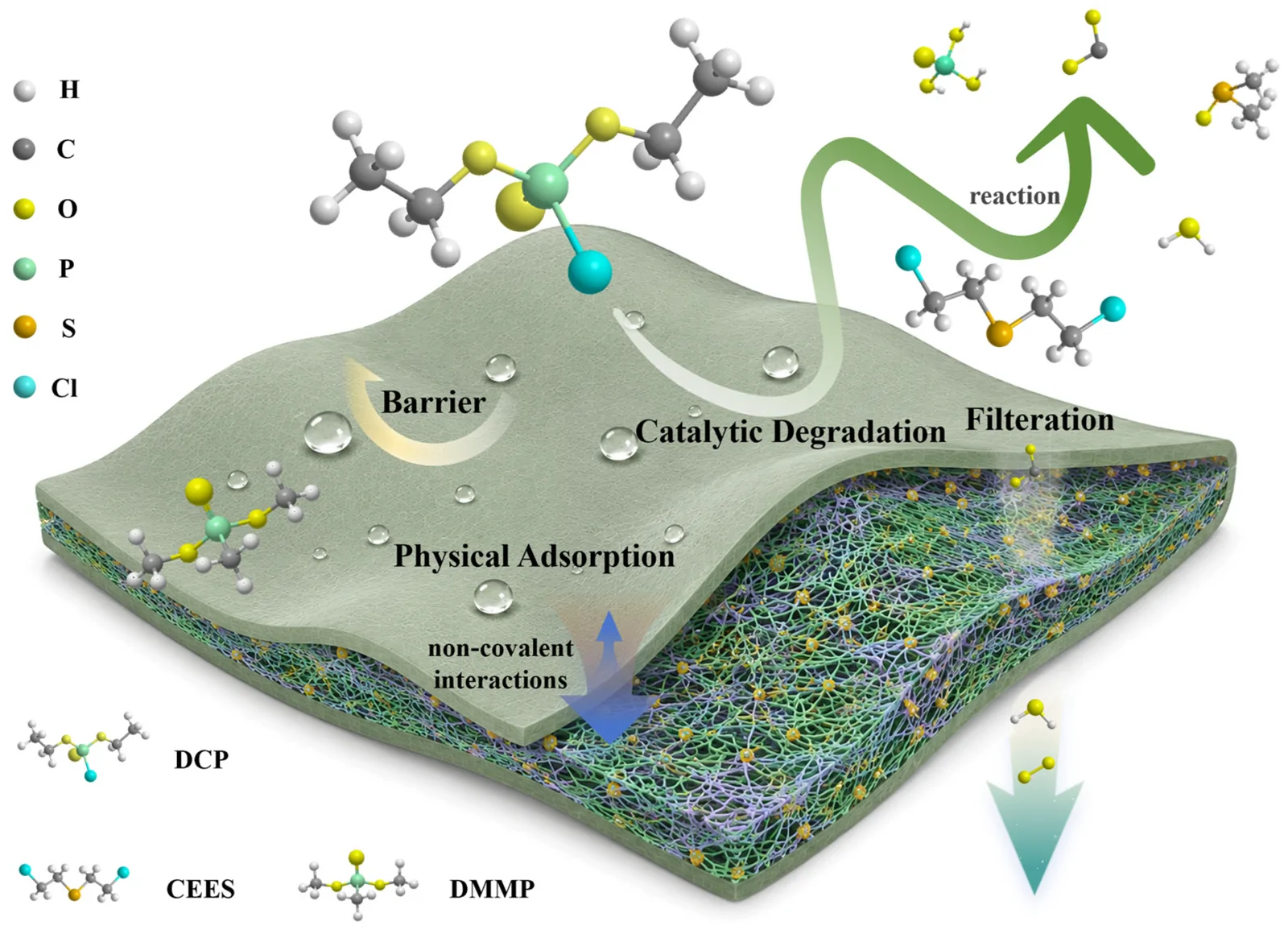
Schematic illustration of the major protection mechanisms of polymeric materials against CWAs, including barrier protection, physical adsorption, filtration, and catalytic degradation. The arrows schematically indicate the transport, adsorption/interactions, interception, and catalytic degrada-tion of CWA molecules within or on the polymeric materials.

**Figure 4:**
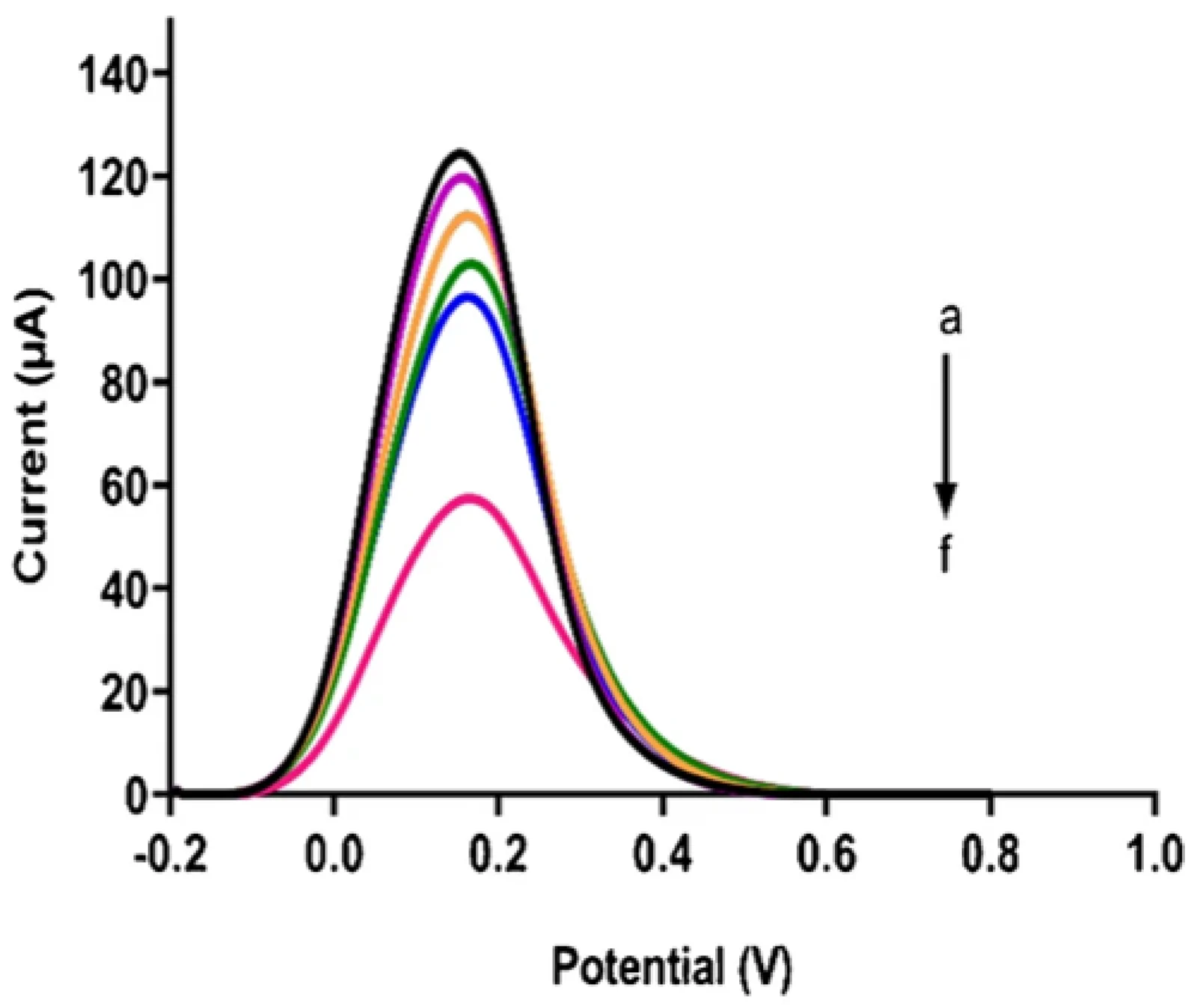
Differential pulse voltammograms of the 4-ABA@EMPA/MIP/GCE sensor after rebinding of different EMPA concentrations (0.1–2.5 nM) in standard solution. The MIP film recognizes EMPA primarily through hydrogen bonding between the –NH_2_/–COOH groups of poly(4-ABA) and the P=O/–OH groups of EMPA. This figure is cropped from Figure 4b in ref. [49] and is used under the Creative Commons Attribution 4.0 International License (CC BY 4.0). Cop-yright © 2024 The Author(s), published by Springer Nature.

**Figure 5:**
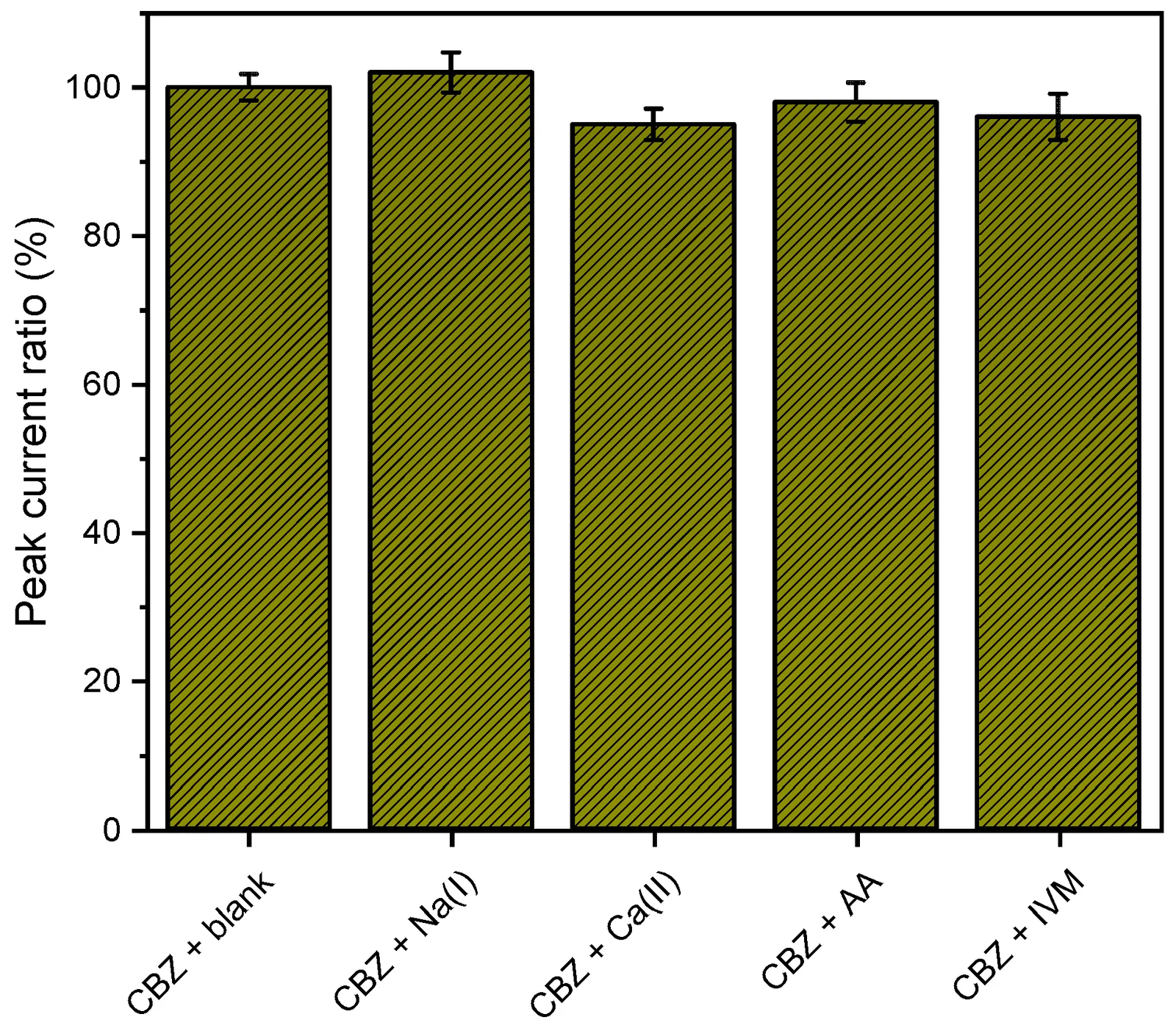
Selectivity of a molecularly imprinted polymer (MIP)/multi-walled carbon nano-tube (MWCNT) electrode toward carbendazim in the presence of various interferents. This figure is reproduced from Figure 9 in ref. [57] under the Creative Commons Attribution 4.0 International License (CC BY 4.0). Copyright © 2024 The Author(s), published by MDPI. The image is used without modification.

**Figure 6:**
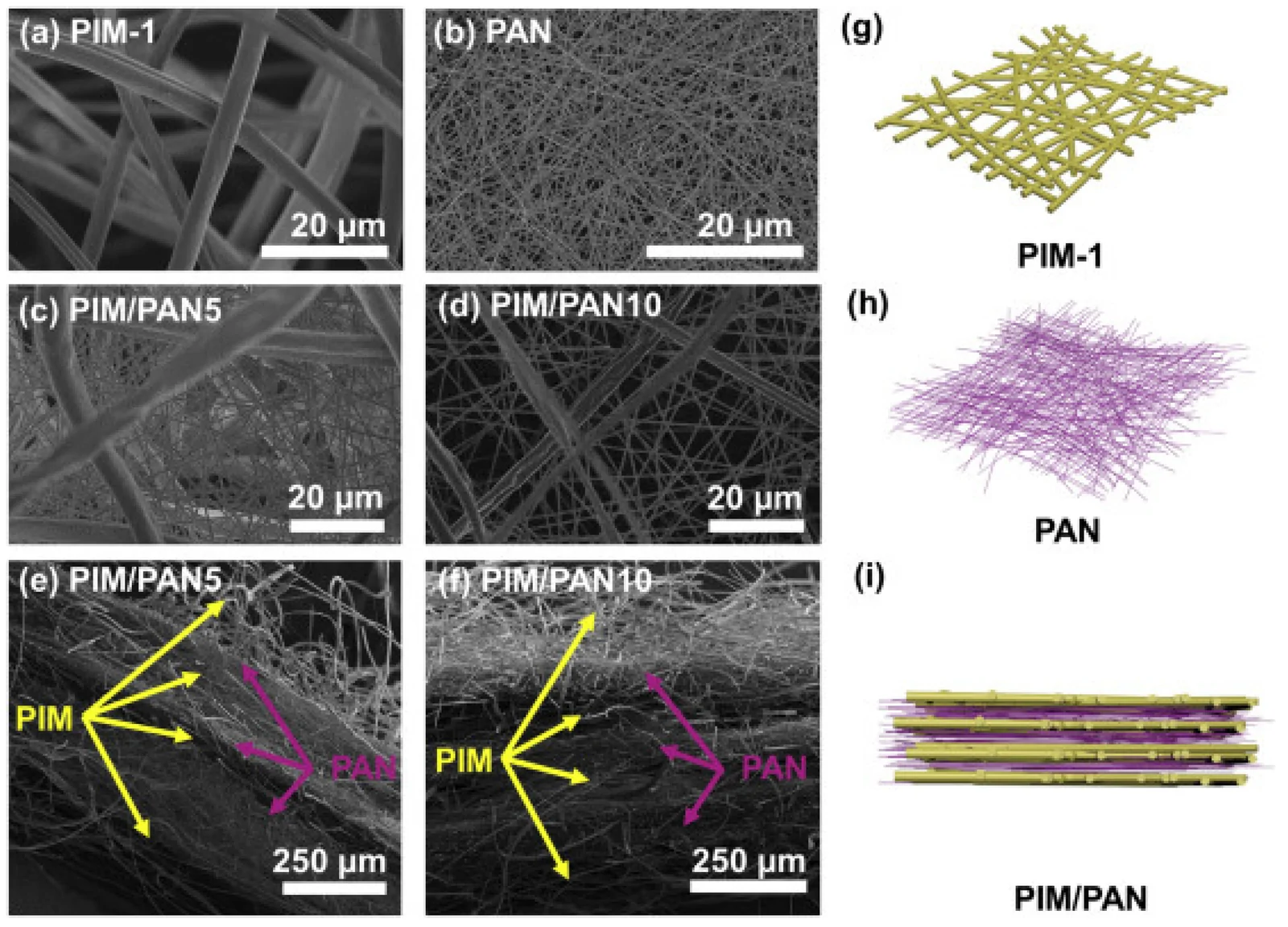
Top view SEM image of (**a**) PIM-1, (**b**) PAN, (**c**) PIM/PAN5, and (**d**) PIM/PAN10 fibers. Cross-section SEM image of (**e**) PIM/PAN5 and (**f**) PIM/PAN10 fibers. Schematics of (**g**) PIM-1 fiber web, (**h**) PAN fiber web, and (**i**) cross-section of PIM/PAN fiber web. PIM, polymer of intrinsic microporosity; PAN, polyacrylonitrile; MOF, metal-organic framework; SEM, scanning electron microscope [34].

**Table 1:** Representative examples of conducting polymer-based chemiresistive sensors relying on the electron transfer mechanism.

Device Type	Material System	Modification/Preparation Strategy	Target Analyte	Key Performance Metrics	Application Scenario
Room-temperature gas-phase chemiresistive device	Black phosphorus/carbon nanotube composite polypyrrole gas-sensitive film Polypyrrole/rGO hydrogen-bonding modified film Carbon nanofiber-supported polypyrrole flexible film	BP and CNT mixed paste, MEMS silicon chip coating with heating assistance In-situ growth of polypyrrole on rGO, hexafluoroisopropanol modification for enhanced selectivity Electrospun nanofiber substrate	DMMP	Linear range12.1–242 nM, reversible regeneration, response 50–75 s Room-temperature response amplitude 12.9%, resistance to low-level alcohol interference 0.004 nM ultra-low limit of detection (LOD), wearable	Real-time monitoring of toxic gas in confined spaces [41] Field screening of atmospheric toxic agents Protective clothing patch warning [43]
Liquid-phase electrochemical rigid electrode	Polyaniline boronic acid/DNA-CNT/nitrogen-doped graphene ternary composite electrode	Glassy carbon electrode with multi-layer nano-modification and electropolymerization	Dopamine	LOD 14 nM, effective shielding of ascorbic acid	Biological samples (serum, cerebrospinal fluid) [44]
Liquid-phase electrochemical porous electrode	Polyaniline-zirconia ordered macroporous material	Conductive polyaniline loaded on porous zirconia	Methyl parathion	LOD 0.228 nM, tolerance to ionic impurities in water	Surface water, agricultural water [45]
Liquid-phase enzymatic electrochemical electrode	Polyaniline/carbon nanotube-immobilized acetylcholinesterase	Carbon nanotube network immobilized hydrolase	Chlorpyrifos	LOD 0.025 nM, long-term stability	Fruit and vegetable extracts, water samples [46]
Liquid-phase enzymatic electrochemical array	CNT-wrapped polypyrrole-polyaniline copolymer	Enzyme immobilized on dual-conductive interpenetrating network	Malathion	LOD 3.03 nM, reusable enzyme	Simultaneous detection of multiple pesticide residues in water samples [47]

**Table 2:** Representative examples of polymer-based sensing materials relying on fluorescence quenching (ratiometric fluorescence) mechanism.

Material System	Sensitive Material	Target	LOD	Response Time
TADF PT/PPT film	Phenoxazine–terpyridine D–A TADF polymer	DCP (simulant)	PT:3 ppb; PPT:2.9 ppb	<5 s [53]
UiO-66-NH_2_@pyrene film	MOF-confined dual-emission monomer; acid-induced quenching	DCP (simulant)	ppt level (ratiometric)	4–6 s [54]
Au@Cu:Tb-MOF-MIP electrochemiluminescence (ECL) film	Au–Cu doped lanthanide MOF + MIP; ECL	Chlorpyrifos	0.029 pg/mL	12 s [56]
C-dots/polymer film	Carbon dots in conjugated polymer matrix; quenching	DCP (simulant)	ppb level	6–9 s [51]
GQD-HBA dual-mode film	graphene quantum dot (GQD) + HFIP polymer; optical + electrochemical	DMMP (simulant)	Fluorescence + resistance dual-signal	7–10 s [42]
Naphthalimide-grafted polymer	Non-conjugated backbone with fluorophore side-chains	DCP (simulant), CEES (simulant)	Wavelength blue-shift discrimination	8–11 s [52]

**Table 3:** Barrier strategies for protection against CWAs and representative CWA simulants.

Strategy Category	Representative Material System	Core Mechanism	Key Structural Parameters	Key Protective Performance	Ref.
Molecular sieving	LPEI/GO layered composite membrane	Size exclusion of GO nanosheets combined with hydrogen-bond adsorption by amine groups on polymer chains	Interlayer free pore size: 0.22 nm	DMMP barrier efficiency: 72.65%; water/DMMP selectivity: 66.6	[60]
Molecular sieving	ZIF-8/SGO hybrid membrane	Precise molecular sieving by intrinsic ZIF-8 pores plus tortuous diffusion path provided by GO nanosheets	ZIF-8 aperture size: 3.4 Å	97% reduction in DMMP permeability; water/DMMP selectivity: 312	[61]
Dense crosslinking	Fluorosilicone-modified epoxy acrylate coating	Dense crosslinked network suppresses molecular diffusion; migrated fluorinated segments construct a super-liquid-repellent surface	Maximum crosslinking density: 3921 mol·m^−3^	DMMP barrier efficiency: 99.96%; CEES barrier efficiency: 100%	[62]
Fibrous interception	PI electrospun nanofiber membrane with PDMS coating	Three-dimensional fibrous network physically intercepts aerosols; PDMS overlayer provides hydrophobic barrier against liquid droplets	Average fiber diameter: 500 nm	PM_0.3_ filtration efficiency: 96.13%; air permeability: 265.44 mm·s^−1^	[64]
Interception–adsorption synergy	ECM-mimetic TPU/FPU fibrous membrane with SBS-AC matrix	Elastic fibrous scaffold serves as structural barrier; activated carbon in the matrix enables physical adsorption of toxic molecules	Hierarchical fiber–matrix composite structure	CEES permeation rate: 0.00214 μg·cm^−2^·min^−1^; stable performance after 1000 tensile cycles	[63]

**Table 4:** Catalytic degradation strategies for CWAs.

Strategy Category	Representative System	Active Sites/Core Modification	Testing Condition	Target Simulant	t_1_/_2_ or Conversion	Reusability	Ref.
Pore microenvironment engineering	MOF-808@MEA	Amine solid-base sites + hydroxyl moisture-trapping sites	60% RH, no buffer	DMNP	82.1% conversion at 1 h	—	[67]
Pore microenvironment engineering	UiO-66-NH_2_@DA	Catechol–amino amphoteric buffering sites	Pure aqueous solution	DMNP	t_1_/_2_ = 7.0 min	—	[68]
Pore microenvironment engineering	MOF-808/PVDF–PVP–imidazole MMM	Imidazole basic catalytic sites + PVP moisture-storage sites	98% RH, solid-state	DMNP	t_1_/_2_ = 5 min	—	[69]
Pore microenvironment engineering	FeCoO_x_@PAN@MOF-808-IMDC	IMDC-functionalized Zr_6_ oxo clusters	Pure aqueous solution	DMNP	t_1_/_2_ = 6.6 s	—	[75]
Pore microenvironment engineering	Cu single-atom supported UiO-66-NH_2_	Cu–N_2_ coordinated single-atom sites	Pure aqueous solution	DMNP	t_1_/_2_ ≈ 2 min; ~8-fold increase in initial rate	—	[71]
Macroscopic shaping	UiO-66-NH_2_@PES composite beads	Exposed Zr_6_ cluster active sites	Pure aqueous solution	DMNP	t_1_/_2_ = 5.09 min	Stable over 3 cycles	[72]
Macroscopic shaping	Dual-MOF functionalized PI nanofabric	Dual-MOF synergistic catalytic sites	Pure aqueous solution	DMNP/CEES	99.5% DMNP @ 20 min; 99.6% CEES @ 24 h	>96% after 5 cycles	[73]
Macroscopic shaping	PAN@Zr(OH)_4_@MOF-808 core–shell nanofiber	Outer-layer MOF hydrolysis sites + inner-layer hydroxide oxidation sites	Pure aqueous solution	DMNP/CEES	t_1_/_2_ (DMNP) = 1.19 min; CEES permeation < 1%	Stable performance	[74]
3D scaffold integration	MOFs/MIPs@graphene oxide aerogel	Zr_6_ cluster catalytic sites + carboxyl self-buffering skeleton	Pure aqueous solution, no exogenous buffer	DMNP	t_1_/_2_ = 3.11 min	>93.5% after 10 cycles	[12]

## Data Availability

No new data were created or analyzed in this study. Data sharing is not applicable.

## Dual-Use Research Statement

The authors have included the following statements in our paper entitled “Polymers for Detecting or Protecting Against Chemical Warfare Agents: Recent Advances and Future Perspectives”.

## Explanation of Potential Risks

This review discusses polymer-based materials for the detection, protection, adsorption, filtration, and catalytic detoxification of chemical warfare agents (CWAs). The work is intended exclusively to support defensive applications, including the development of sensing, protective, decontamination, and detoxification materials. The manuscript does not involve the synthesis, preparation, weaponization, or optimization of actual CWAs, nor does it provide experimental protocols for their production or harmful use.

## Evaluation of Benefits to the General Public

The research provides a systematic overview of polymer-based materials and their structure–property relationships for CWA detection and protection. The findings are intended to facilitate the development of safer sensing, protective, and decontamination technologies and to contribute to materials science, environmental safety, and public protection.

## Compliance with Laws and Regulations

The authors recognize the potential dual-use implications associated with research concerning CWAs and have conducted this work in accordance with applicable national and international laws, regulations, and institutional requirements concerning chemical safety, dual-use research, and the responsible conduct of research.

## Abbreviations

The following abbreviations are used in this manuscript:

Abbreviation	Full Term
AC	activated carbon
BET	Brunauer–Emmett–Teller
BHPHFP	2,2-bis(4-hydroxyphenyl)hexafluoropropane
CEES	2-chloroethyl ethyl sulfide
CMP	conjugated microporous polymer
CNT	carbon nanotube
COFs	covalent organic frameworks
CuO	copper(II) oxide
CWAs	chemical warfare agents
DCP	diethyl chlorophosphate
DFT	density functional theory
DMCP	dimethyl chlorophosphate
DMF	N,N-dimethylformamide
DMMP	dimethyl methylphosphonate
DMNP	dimethyl 4-nitrophenyl phosphate
ECL	electrochemiluminescence
ECM	extracellular matrix
EDA	ethylenediamine
FPU	fluorinated polyurethane
FRET	fluorescence resonance energy transfer
GB	sarin
GD	soman
GO	graphene oxide
GQD	graphene quantum dot
HD	sulfur mustard
HFIP	hexafluoroisopropanol
HOMO	highest occupied molecular orbital
HOFs	hydrogen-bonded organic frameworks
ICT	intramolecular charge transfer
IMDC	4,5-imidazoledicarboxylic acid
IMS	ion mobility spectrometry
ISIL	Islamic State of Iraq and the Levant
LOD	limit of detection
LPEI	linear polyethyleneimine
LUMO	lowest unoccupied molecular orbital
MEA	monoethanolamine
MEMS	micro-electro-mechanical system
MIPs	molecularly imprinted polymers
MOFs	metal–organic frameworks
PAN	polyacrylonitrile
PANI	polyaniline
PDA	polydiacetylene
PCDA	10,12-pentacosadiynoic acid
PDMS	polydimethylsiloxane
PEDOT	poly(3,4-ethylenedioxythiophene)
PEN	poly(arylene ether nitrile)
PET	photoinduced electron transfer
PES	polyethersulfone
PI	polyimide
PIMs	polymers of intrinsic microporosity
PPy	polypyrrole
PTh	polythiophene
PVDF	poly(vinylidene fluoride)
PVP	polyvinylpyrrolidone
QCM	quartz crystal microbalance
QEPAS	quartz-enhanced photoacoustic spectroscopy
RH	relative humidity
rGO	reduced graphene oxide
SBS	styrene–butadiene–styrene block copolymer
TADF	thermally activated delayed fluorescence
TPU	thermoplastic polyurethane
VX	VX nerve agent
WVTR	water vapor transmission rate
XPS	X-ray photoelectron spectroscopy
ZIF-8	zeolitic imidazolate framework-8
MMM	mixed-matrix membrane
PAK	fluorosilane-epoxyacrylate ternary prepolymer
